# BBB-aware stimuli-responsive and biomimetic nanomedicines for glioblastoma

**DOI:** 10.1186/s41016-026-00438-6

**Published:** 2026-07-01

**Authors:** Sana Javaid, Wenqi Song, Sajid Ali, Wei Hou, Xueqiong Su, Hao Wang, Yujun Song

**Affiliations:** 1 Central for Modern Physics Technology, Center for Green Innovation, School of Mathematics and Physics, University of Science and Technology Beijing, 30 Xueyuan Road, Haidian District, Beijing, 100083 China; 2 School of Physics and Optoelectronic Engineering, Beijing University of Technology, Beijing, 100124 China; 3 Beijing Tiantan Hospital, Capital Medical University, No. 119, Nan Sihuan West Road, Fengtai District, Beijing, 100070 China

**Keywords:** Glioblastoma, Blood–brain barrier, Blood–brain tumor barrier, Nanomedicines, Stimuli-responsive drug delivery systems, Biomimetic nanoparticles, Magnetic hyperthermia, Photothermal and photodynamic therapy

## Abstract

Glioblastoma (GBM) poses a tremendous challenge because it causes substantial morbidity and mortality. Treatment remains constrained by the tightly regulated blood–brain barrier (BBB) and the heterogeneous blood–brain tumor barrier (BBTB), which together severely limit drug delivery to tumor tissue. Nanomaterial-based drug delivery systems offer an opportunity to overcome the short half-life, low bioavailability, and poor BBB penetration that restrict conventional GBM therapeutics. Nanotechnology also provides safe, effective, and targeted drug delivery systems that enhance penetration, stability, and therapeutic efficacy. This review discusses biomaterials-based nanomedicine platforms for GBM, with a focus on lipid-based carriers, polymeric nanoparticles, dendrimers, inorganic nanomaterials, and biomimetic nanosystems designed to interact with the BBB/BBTB. We summarize how passive and active brain-targeting strategies are combined with endogenous and exogenous stimuli-responsive designs (pH/redox sensitivity, magnetic hyperthermia, photothermal/photodynamic therapy, and ultrasound-triggered systems) to enhance intratumoral accumulation and anti-GBM efficacy. Overall, this review discusses recent advances in BBB-aware, stimuli-responsive, and biomimetic nanomedicines for GBM, and outlines their therapeutic potential alongside persistent challenges in safety, large-scale manufacturing, and clinical translation.

**Graphical Abstract:**

Illustration of ligand-functionalized nanomedicines crossing the BBB using magnetic and electric responsive stimuli.

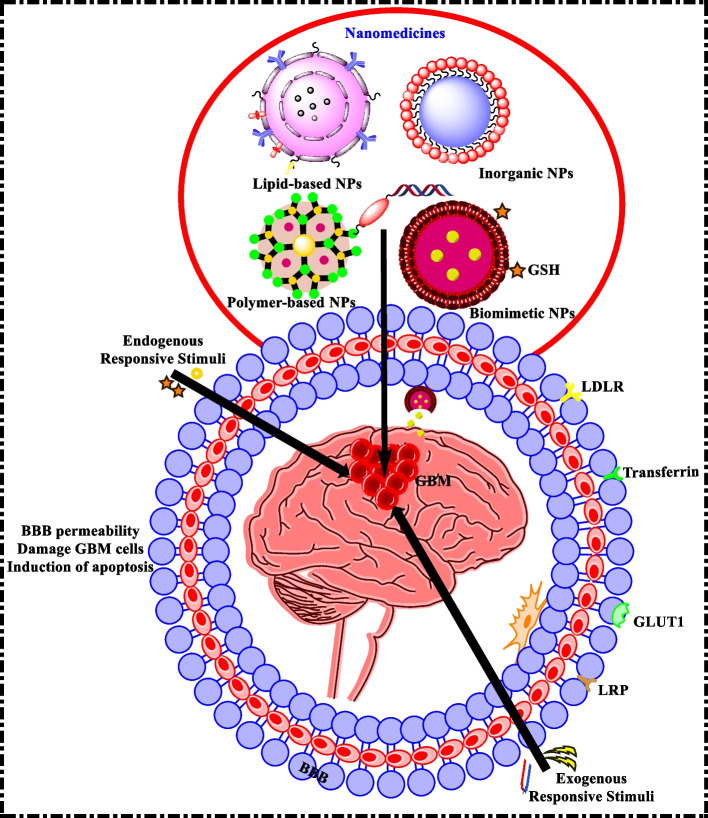

## Background

Glioblastoma multiforme (GBM), a WHO grade IV diffuse astrocytic tumor, is the most common and aggressive primary malignant brain tumor in adults. It accounts for ~ 45% of malignant brain and CNS tumors [1] and carries a median overall survival of only 14–15 months despite standard-of-care-therapy [2–4]. Histologically, GBM shows high cellular density, pleomorphic and distorted nuclei, microvascular proliferation, necrosis, and marked invasiveness. Molecularly, recurrent alterations such as TERT promoter activation, epidermal growth factor receptor (EGFR) amplification/EGFRvIII, and chromosome + 7/− 10 are prominent drivers of proliferation, genomic instability, and treatment resistance [1, 5]. Hypoxia is another hallmark; stabilization of HIF-1α upregulates angiogenic factors, reprograms glucose and fatty-acid metabolism, shapes the tumor-immune microenvironment, and promotes autophagy-mediated survival, all of which contribute to GBM aggressiveness [6].

Current standard management consists of maximal safe surgical resection followed by radiotherapy with concomitant and adjuvant temozolomide (TMZ) [7–10]. However, durable benefits are rare. Radiotherapy unavoidably affects the surrounding normal brain and can cause neurocognitive decline, including clinically meaningful cognitive impairment in some patients. Meanwhile TMZ, the first-line chemotherapeutic agent, is limited by rapid development of chemoresistance and by poor drug penetration across the blood–brain barrier (BBB) [4, 11–15].

As a result, inefficient drug delivery, limited targeting specificity, systemic toxicity, and the inability to monitor treatment response in real time remain major challenges in GBM management. It is estimated that ~ 98% of small-molecule drugs and nearly all biologics do not cross the intact BBB at therapeutic levels [16].

The BBB, composed of closely connected brain microvascular endothelial cells supported by pericytes and astrocytes, tightly regulates molecular exchange between the blood and CNS. While this neuroprotective role is essential, it severely restricts most anticancer agents from reaching intracranial tumors. To overcome these barriers, recent research has focused on nanotechnology-based therapeutic strategies. Multifunctional nanocarriers are being engineered to bypass or exploit BBB/blood–brain tumor barrier (BBTB) transport pathways, provide brain- and tumor-targeted delivery, enable controlled or stimuli-responsive drug release, and enhance retention within GBM lesions [17, 18].

Nanomaterial-based drug delivery systems (NDDSs) have therefore emerged as a promising strategy to address these barriers. Engineered nanoparticles (NPs) can be designed to enhance brain and tumor-specific delivery, promote controlled drug release, and, in some cases, enable the conversion of external physical fields (light, magnetic, ultrasound, or heat) into local cytotoxic effects [15, 17–19].

Common platforms include lipid-based nanoparticles (liposomes, solid lipid nanoparticles, lipid nanocapsules), polymeric nanoparticles, dendrimers, and inorganic nanomaterials (e.g., gold and iron oxide) [20, 21]. These carriers extend circulation time, improve solubility, and enhance tumor deposition relative to free drugs, while surface functionalization (e.g., with peptides, antibodies, or aptamers) and stimuli-responsive designs allow for active targeting and on-demand release [22, 23]. Beyond small-molecule chemotherapeutics, NDDSs can encapsulate proteins, antibodies, and nucleic-acid therapeutics (siRNA, miRNA, mRNA, CRISPR/Cas systems), thereby broadening anti-GBM mechanisms to include DNA damage, oncogene silencing, immune activation, and epigenetic modulation [24, 25]. Drawing on principles from biomaterials science, physical-field–responsive nanomedicines have opened additional avenues for GBM therapy. Inorganic cores such as gold or iron oxide enable the conversion of external fields near-infrared (NIR) light, alternating magnetic fields, ultrasound, or ionizing radiation into heat (photothermal therapy, PTT; magnetic hyperthermia) or reactive oxygen species (photodynamic and sonodynamic therapy, PDT/SDT), thus amplifying DNA damage, apoptosis, immunogenic cell death, and vascular disruption specifically within the tumor [24, 26, 27]. Recent advancements in nanotechnology have led to reactive oxygen species (ROS)-based nanotherapy, a novel strategy that effectively targets glioma by generating significant amounts of ROS within these cells [28, 29]. In parallel, biomimetic nanosystems, including cell membrane–coated nanoparticles, lipoprotein-mimetic carriers, and nanoparticle hydrogel hybrids seek to exploit endogenous transport pathways, immune evasion, and homotypic targeting to improve BBB transit and localization within infiltrative GBM margins.

For GBM applications, such design principles are particularly important: smaller, neutrally or slightly negatively charged NPs are better candidates for combining adequate BBB penetration with eventual systemic elimination, potentially reducing long-term toxicity. Our group and others have emphasized that NPs with sizes below specific thresholds (e.g., < 6 nm for efficient renal clearance, and in some designs < 3 nm to favor BBB transit) hold promise for achieving both effective delivery and safe elimination, although these thresholds may vary across materials and surface chemistries [30, 31].

Clinically, translation is underway. The iron oxide–based NanoTherm® system is approved in Europe for intratumoral magnetic hyperthermia in recurrent GBM, in combination with external beam radiotherapy, and has shown manageable safety with encouraging survival signals in small series [32]. A phase 0 trial of NU-0129, a gold-based spherical nucleic acid (SNA) targeting BCL2L12, provided proof that systemically administered RNAi nanodrugs can cross the human BBB, accumulate in GBM tissue, and achieve target knockdown [33]. Systematic clinical analyses suggest that nanotherapies can modestly extend survival and improve quality of life with acceptable toxicity, while highlighting the need for more standardized, mechanism-focused formulations and trials [34–36].

In this review, we first outline the current clinical and scientific challenges in GBM therapy, including the roles of the BBB, chemoresistance, and limited therapeutic specificity. We then discuss nanomedicine-based strategies developed to overcome these barriers, with a particular focus on anti-GBM mechanisms: how nanodrugs and nanoparticle-mediated physical-field therapies (e.g., magnetic hyperthermia, PTT, PDT, SDT) kill GBM cells and interact with the BBB/BBTB. We summarize brain-targeting strategies (passive, active, and cell-mediated) and detail endogenous and exogenous stimuli-responsive nanodrugs, particularly those integrated with heat, light, magnetic fields, or ultrasound. Finally, we highlight biomimetic nanosystems and other advanced biomaterial platforms, and discuss current clinical efforts and future directions, including AI-assisted design and mechanism-guided optimization of GBM nanomedicines.

## Biological barriers in glioblastoma relevant to nanomedicine

### Blood–brain barrier

**Fig. 1:**
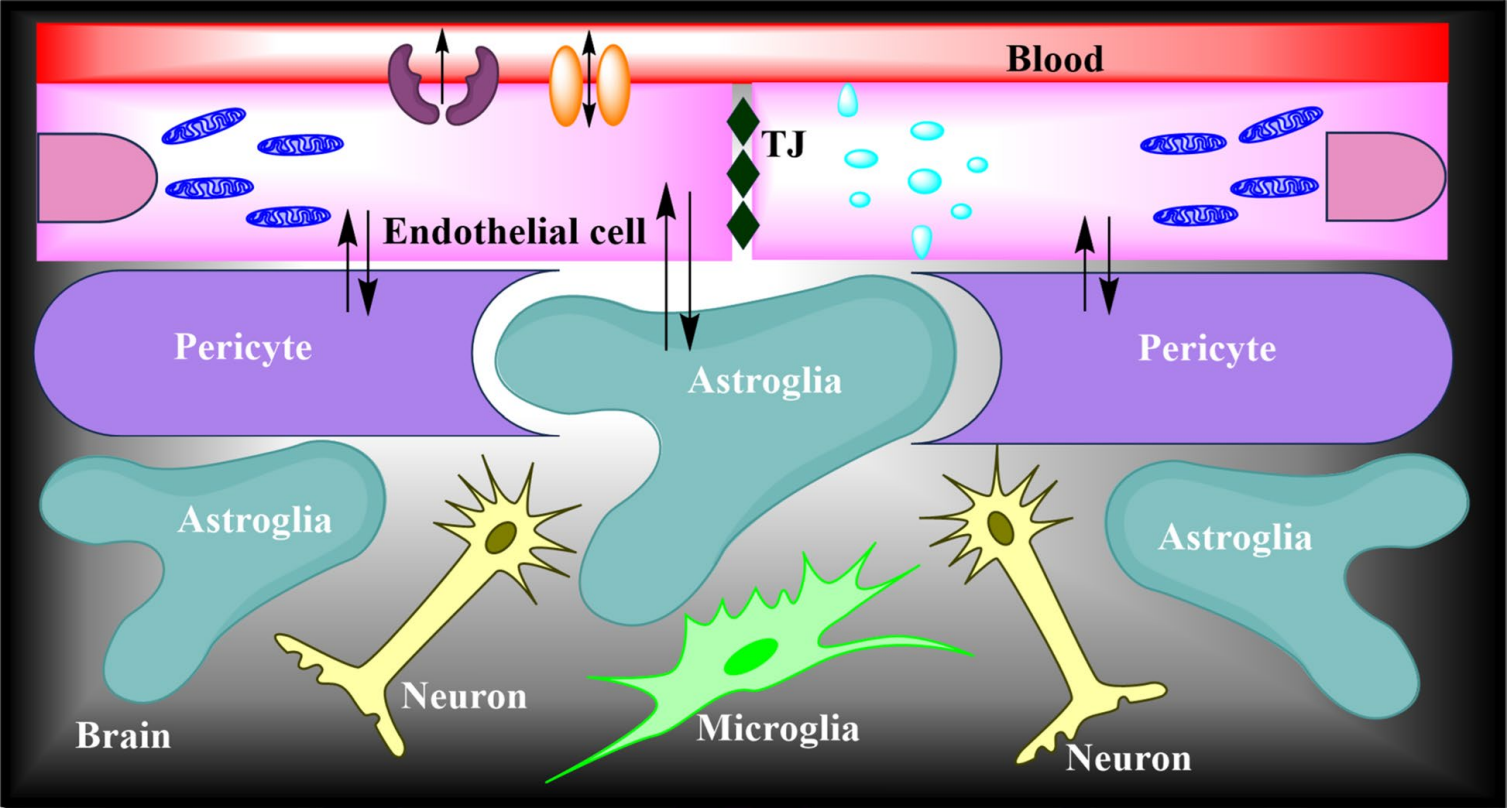
Schematic representation of the neurovascular units that form the BBB. BMECs are closely associated with pericytes and astrocytic end-feet, while neurons and microglia modulate barrier function [37]

The BBB is a highly specialized interface between the systemic circulation and the CNS parenchyma. It is formed by brain microvascular endothelial cells (BMECs) connected by continuous tight junctions (TJs) and supported by pericytes, astrocytic end-feet, microglia, and neurons, collectively constituting the neurovascular unit (Fig. 1).

BBB endothelial cells express ATP-binding cassette (ABC) efflux transporters such as P-glycoprotein (ABCB1) and breast cancer resistance protein (ABCG2), which actively extrude many xenobiotics and chemotherapeutics back into the bloodstream, as well as solute carrier transporters that facilitate nutrient influx (e.g., GLUT1 for glucose, LAT1 for neutral amino acids). Together with metabolic enzymes and basement membrane components (e.g., laminin-α5), this transporter/enzyme network shapes the pharmacokinetic and pharmacodynamic profiles of CNS-directed drugs [37–40].

**Fig. 2:**
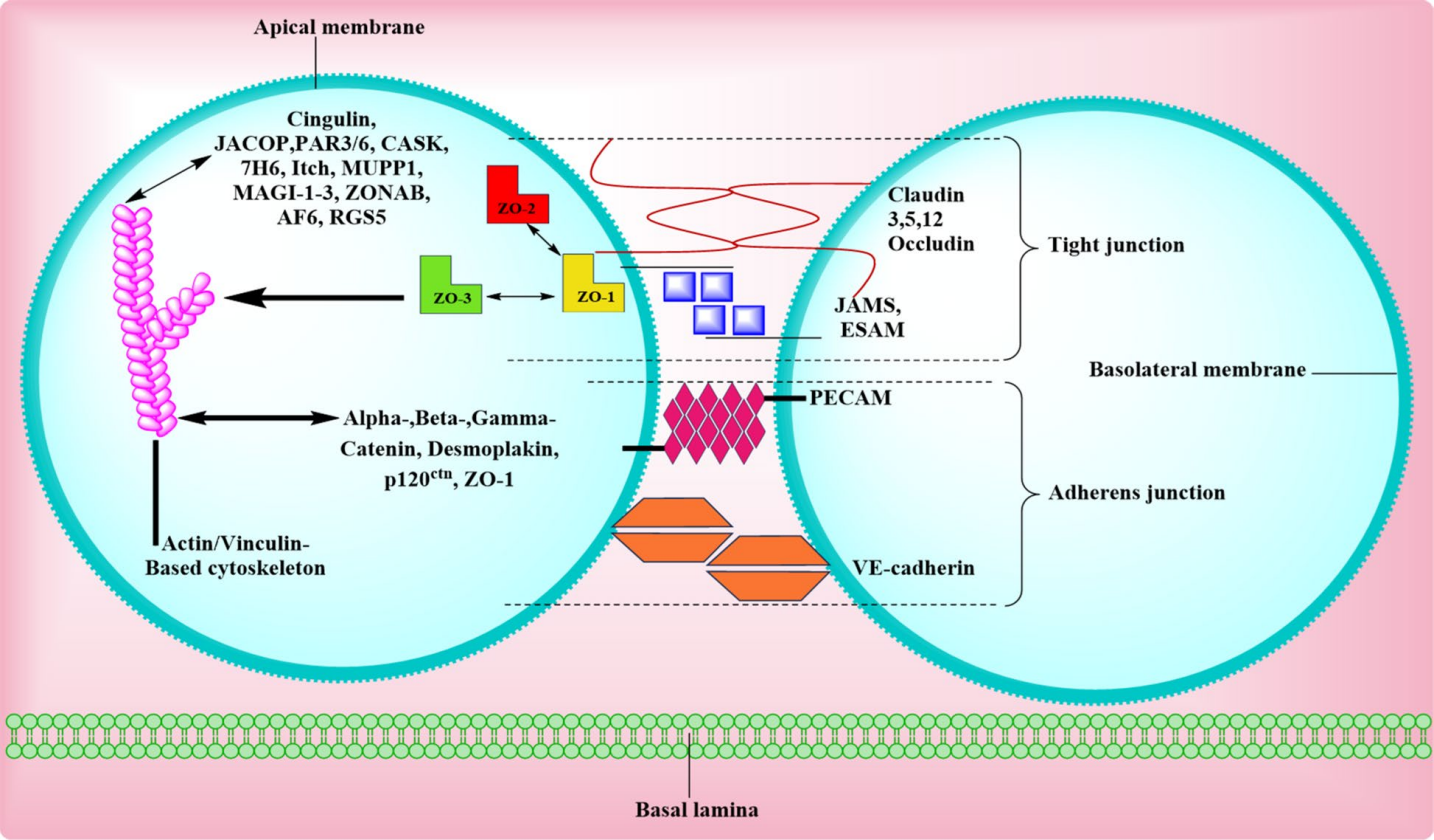
Simplified molecular organization of endothelial tight junctions, highlighting claudins, occludin, and associated scaffolding proteins [41]

At the molecular level, TJ disruption and transporter dysregulation are common in CNS diseases, and the molecular organization of endothelial tight junctions is summarized in Fig. 2. In GBM, tumor-driven angiogenesis and chronic inflammation remodel the vasculature, creating a blood–brain tumor barrier (BBTB) with heterogeneous permeability. Regions within the enhancing tumor core often display leaky, tortuous vessels with reduced TJ integrity, whereas infiltrative margins may retain an almost intact BBB. This “leaky core versus protected invasive front” pattern is a key reason why even BBB-permeant drugs fail to uniformly eradicate GBM [41].

To transiently modulate the BBB/BBTB for drug delivery, noninvasive strategies such as low-intensity focused ultrasound (LIFU) combined with intravenously administered microbubbles have been developed. LIFU induces reversible TJ opening, increasing local uptake of chemotherapeutics, antibodies, and nanomedicines within a confined intracranial volume while limiting systemic exposure. Early clinical and preclinical data in GBM and other brain tumors demonstrate that this approach can safely enhance intratumoral drug levels and prolong survival, making it highly relevant to field-assisted and stimuli-responsive nanomedicine strategies [42–44].

From a biomaterials standpoint, these BBB features define the design space for GBM nanomedicines: nanocarriers must either (i) remain sufficiently small, neutrally or slightly negatively charged, and “stealthy” to exploit subtle BBB/BBTB leakiness, or (ii) actively engage endogenous transport pathways while maintaining structural stability and controlled responsiveness to local or external triggers.

#### Mechanism of BBB transport

A mechanistic understanding of trans-BBB transport is essential for rational GBM nanomedicine design. Nanoparticles can exploit several physiological transport routes:

- Receptor-mediated transcytosis (RMT). Ligands on the NP surface bind endothelial receptors (e.g., transferrin receptor, insulin receptor, LRP1, LDLR, lactoferrin receptor), triggering endocytosis and vesicular shuttling across the BBB.
- Carrier-mediated transport. Small-molecule prodrugs or surface-grafted moieties (e.g., glucose, amino acids) hijack nutrient transporters such as GLUT1 or LAT1.
- Adsorptive-mediated transcytosis. Cationic or cell-penetrating peptide modified NPs interact electrostatically with negatively charged endothelial surfaces, inducing non-specific uptake.

Among these, RMT is the most widely exploited in GBM nanotherapy. Examples include Angiopep-2–decorated polymeric NPs targeting LRP1, transferrin (Tf) modified liposomes, and apolipoprotein-mimetic peptide-functionalized gold NPs [45–48], all of which show improved BBB crossing and intratumoral delivery relative to untargeted carriers (Tables 1 and 2).

**Table 1:** Nanocarriers for glioma therapy via exploiting BBB transport

Drug	Nanocarrier	Function	References
Daunomycin	Liposomes	Anti-transferrin (Tf) receptor antibody	[49]
Temozolomide (TMZ)	Polymeric NPs	Angiopep-2	[50]
Paclitaxel (PTX)	PEG-PLA micelles	EGFR/EGFRvIII targeting peptide	[51]
Doxorubicin (DOX)	PEGylated liposomes	Glutathione (GSH)	[52]
Docetaxel	Solid lipid nanoparticles (SLN)	HBA	[53]
Temozolomide (TMZ)	Olive oil NPs	Lactoferrin (Lf)	[54]

**Table 2:** Examples of receptors or carrier-mediated BBB targeting strategies for GBM and/or glioma nanotherapy

**Target/Carrier**	**Ligand**	**Disease**	**Therapeutic** agent	**Nanocomposite**	**Effects**	**Ref**
TfR	T7	Glioma	Vincristine sulphate	Low-density lipoprotein particles	Demonstrating increased localization in gliomas and a positive anti–glioma effect both in vivo and in vitro	[46]
TfR	T10	Glioma	Doxorubicin	Versatile hollow COF nanospheres	T10 corona enables cascade-targeting glioma drug delivery across the BBB	[55]
TfR	T12	Glioma	Vinblastine	Liposomes	RMT and subsequent uptake by glioma cells and GSCs, increasing necrosis	[48]
TfR	OX26	Brain Cancer	Cisplatin	PEGylated liposome	Greatly improved cellular uptake; 1.7-fold increase in survival in rats with brain tumors	[56]
LDLR/LRP1	Angiopep-2	GBM	Cisplatin; TMZ	Angiopep-2-functionalized lipid cubosomes	Increased brain accumulation and enhanced toxicity in U87 spherocytes	[57]
LDLR	Pep22	GBM	Paclitaxel	LDLR mediated pep-22-conjugated NPs	Able to cross the BBB and target glioma cells in vitro	[58]
LDLR	ApoB	GBM	AuNPs	LDLR ligand-functionalized gold nanoparticles	Effective targeting of the TME significantly improved therapeutic efficacy	[59]
LDLR/ApoE rec	ApoE	GBM	Ph NPs	ApoE-Ph NPs	Targeting gliomas and promoted effective BBB crossing	[60]
Integrins	cRGD	GBM	TMZ	R‐NKm@NPs	Enhanced NK-cell cytotoxicity and reduced apoptosis; improved anti-GBM effects	[61]
Integrins	cRGD	GBM	mRNA	Biomimetic CaCO₃ NPs	Enhanced GBM targeting after BBB crossing; supports sono-immunotherapy with IL-12 mRNA	[62]
LfR	Lf	GBM	Glycyrrhizin	Lactoferrin-glycyrrhizin (Lf-GL) conjugate	Inhibits angiogenesis and tumor growth; improves GL pharmacokinetics and reduces tumor growth	[63]
EGFR	Cetuximab	GBM	Doxorubicin	Anti-EGFR immunoliposomes (ILs-DOX)	Targeting EGFR-amplified GBM; enhanced uptake and antitumor activity	[64]
EGFR	CFMQ	GBM	TMZ	CFMQ-coupled CS-PLGA NPs	Increased cellular uptake and synergistic enhancement of TMZ efficacy	[65]

The high energetic requirements of the brain are accomplished by a constant supply of glucose, aided by glucose transporters (GLUTs) on the blood–brain barrier. GBM, as a highly proliferative malignant tumor, exhibits a pronounced “Warburg effect,” characterized by extremely vigorous glucose metabolism. To meet this enormous energy demand, GBM cells significantly upregulate GLUTs on their cell membranes, particularly GLUT1 and GLUT3. Notably, these transporters are highly expressed not only on tumor cells but also abundantly on the endothelial cells of the blood–brain barrier (BBB), presenting a dual exploitation opportunity [66, 67]. Glucose or amino acid–decorated nanocarriers that hijack GLUT or LAT1 have shown improved brain uptake and anti-GBM efficacy in preclinical models and represent a complementary strategy to classical receptor-mediated approaches.

## Brain-targeting strategies

**Fig. 3:**
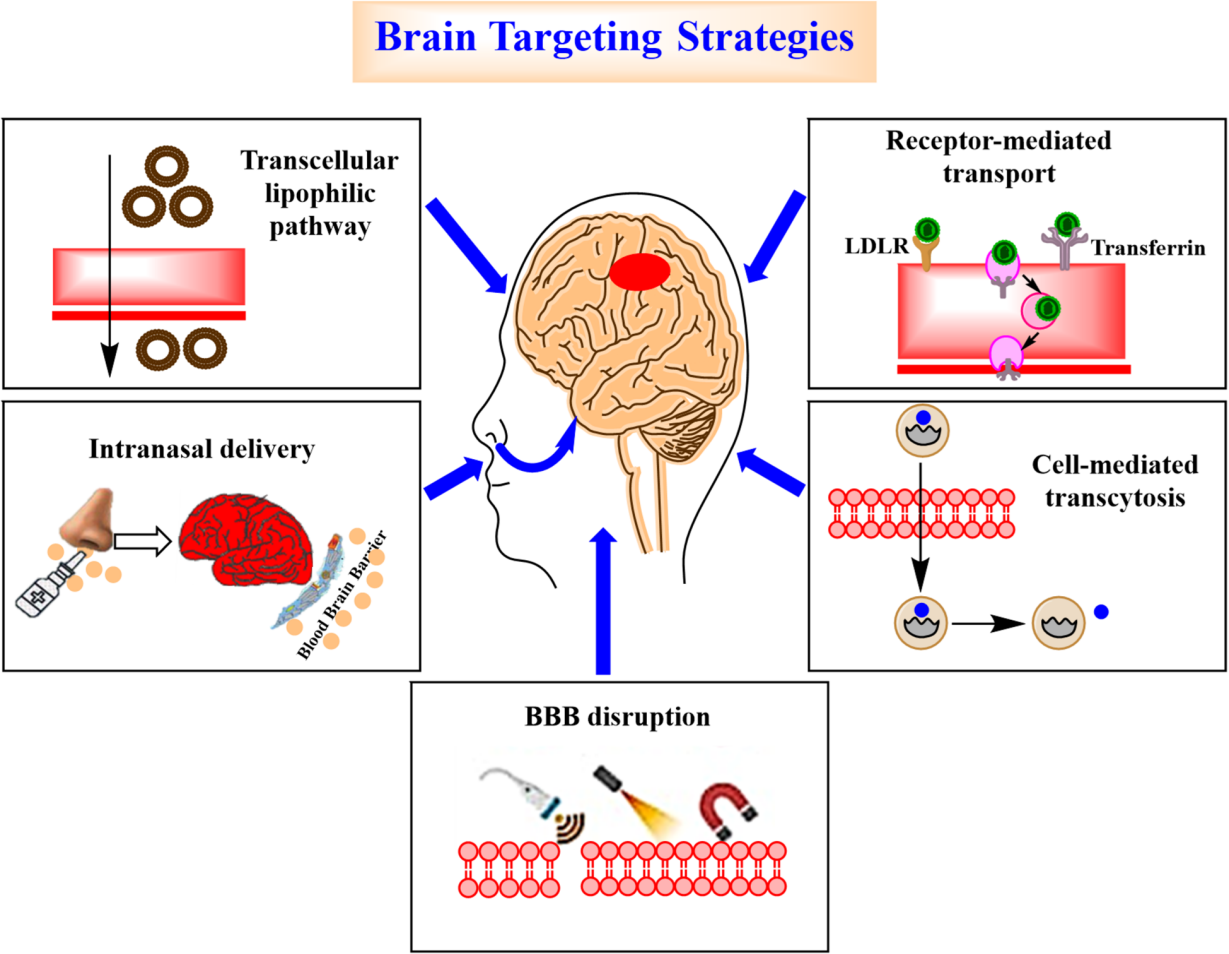
Overview of brain-targeting strategies for GBM nanomedicine

Nanoparticles (NPs) can improve drug delivery across the BBB and into glioblastoma (GBM) through two main, often complementary, approaches: passive targeting and active (ligand-mediated) targeting, frequently integrated with endogenous or exogenous stimuli-responsive designs. These strategies shape how nanodrugs cross the BBB/BBTB, distribute within the tumor, and engage anti-GBM mechanisms such as DNA damage, apoptosis, ferroptosis, and immunogenic cell death. The major brain-targeting strategies used in GBM nanomedicine are summarized in Fig. 3.

### Passive targeting

Passive targeting relies on the inherent physicochemical properties of nanoparticles (NPs), including size, shape, surface charge, and circulation half-life together with tumor-associated vascular abnormalities, rather than on specific ligand–receptor interactions [60]. Confocal and cell fractionation studies in brain endothelial cells indicate that NPs predominantly associate with the plasma membrane and vesicular compartments and enter cells mainly via non-specific endocytosis [60].

**Fig. 4:**
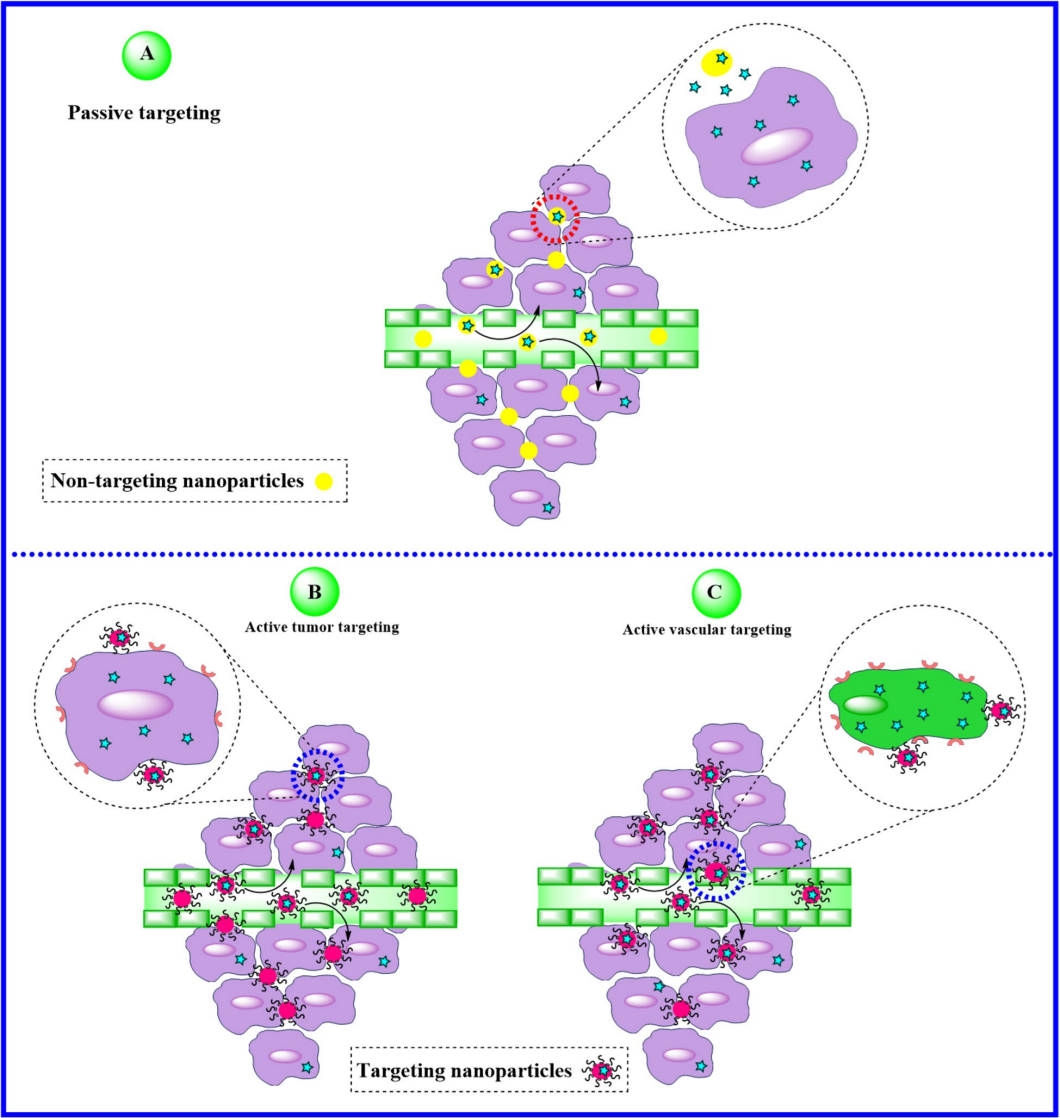
**A** Non-targeted nanoparticles accumulate in tumors and/or inflamed tissues via the enhanced permeability and retention (EPR) effect. **B** Surface-conjugated targeting ligands promote active binding to cell-surface receptors and then internalization via the receptor-mediated endocytosis. **C** In active vascular targeting, nanoparticle accumulation in the vascular wall is driven by specific ligand–receptor interactions rather than by the EPR effect [72]

Surface coatings and polymer “stealth” layers (e.g., PEG and zwitterionic shells) are crucial to reduce opsonization and clearance and to prolong circulation [68]. In GBM, dysregulated angiogenesis and chronic inflammation generate a BBTB with tortuous, dilated vessels and heterogeneous permeability. When tight junctions are disrupted and lymphatic drainage is impaired, an EPR like effect can arise, enabling prolonged retention of long-circulating NPs (Fig. 4A) [69]. However, this effect is highly variable in brain tumors: infiltrative margins often retain an intact BBB, whereas necrotic cores are more permeable, leading to non-uniform NP distribution and residual tumor cell survival [70, 71].

Passively targeted GBM nanomedicines are therefore designed by tuning NP size, surface chemistry, and circulation time. NPs of roughly 10 − 100 nm with hydrophilic, neutrally or slightly negatively charged surfaces and dense stealth coatings can evade rapid clearance by the mononuclear phagocyte system, prolong blood circulation, and better exploit the limited leakiness of the BBTB. Lipid-based systems (liposomes, solid lipid nanoparticles) and polymeric NPs loaded with TMZ, DOX, or PTX have achieved significant growth inhibition in intracranial glioma models via passive accumulation alone. Polysorbate-80 coated PLGA-DOX NPs, for example, show improved uptake and cytotoxicity in A172 glioma cells, likely due to adsorbed apolipoproteins that promote interaction with BBB receptors [68, 73]. Clinically approved oncology nanomedicines such as Abraxane®, Doxil®, and Myocet® rely mainly on passive tumor targeting; although not GBM-specific, they provide proof-of-principle that passive nanocarriers can improve therapeutic index in humans. For GBM, however, the heterogeneity of BBTB permeability and the infiltrative nature of the disease mean that passive targeting alone is usually insufficient, and it is increasingly combined with active targeting and physical-field strategies (e.g., FUS-assisted BBB opening or magnetic guidance) to improve coverage of invasive tumor regions.

### Active targeting

Because the EPR effect in GBM is inconsistent and often weak at tumor margins, active targeting has become central to GBM nanomedicine. Here, NPs are functionalized with ligands that recognize receptors or transporters overexpressed on BBB/BBTB endothelium, GBM cells and glioma stem cell components. Ligand–receptor binding triggers receptor-mediated endocytosis or transcytosis, enabling higher intracellular drug delivery and more robust anti-GBM mechanisms than passive strategies alone (Fig. 4A–C) [72]. Importantly, active targeting not only enhances chemotherapy but also underpins many field-based and stimuli-responsive nanotherapies: sufficient and selective NP accumulation in tumor regions is essential to safely convert light, magnetic fields, or ultrasound into local heat or ROS while sparing healthy brain.

#### BBB/BBTB and GBM-relevant molecular targets

Key BBB/BBTB and tumor targets include:

LRP1/LDLR: Overexpressed on BBB endothelial cells and GBM cells. Angiopep-2 and ApoE/ApoB-mimetic peptides are widely used ligands that enable “dual” targeting of brain endothelium and tumor cells. Angiopep-2–modified polymeric or lipid NPs loaded with PTX or cisplatin achieve higher brain accumulation, enhanced apoptosis, and prolonged survival in orthotopic GBM models compared with non-targeted controls [74–76].

Transferrin receptor (TfR), highly expressed on both BBB and tumor vasculature. Tf-decorated liposomes and PLGA NPs show increased brain uptake and intratumoral drug levels. Anti-TfR antibodies (e.g., OX26) have also been used to shuttle liposomal cargos across the BBB.

Nutrient transporters(GLUT1/GLUT3, LAT1): GBM’s high glycolytic and amino acid demands drive overexpression of glucose and amino acid transporters. Glucose or amino acid–modified NPs can hijack these transporters for selective delivery.

Tumorcell and GSC markers: Targets such as EGFR/EGFRvIII, integrins (αvβ3/αvβ5), CD44, IL-13Rα2, folate receptor, and neuropilin-1 are exploited using antibodies (e.g., cetuximab), peptides (e.g., cRGD), hyaluronic acid, or IL-13 analogues. EGFR-targeted NPs enhance delivery of DOX/TMZ to EGFR-positive GBM and can radiosensitize tumors; integrin-binding cRGD improves NP localization at invasive fronts and angiogenic vessels; CD44-targeted HA-modified NPs deliver drugs or siRNA to glioma stem-like cell (GSCs), helping to suppress recurrence.

Immune and vascular targets: Ligands targeting TAMs/microglia or angiogenic endothelium allow NPs to reprogramme M2-like macrophages toward an M1 phenotype, disrupt tumor vasculature, and modulate immune signaling, indirectly boosting responsiveness to chemo- and field-based therapies.

#### Design and synthesis of targeted GBM nanomedicines

Despite platform diversity, most actively targeted GBM nanomedicines share a modular architecture.

1. Core formation
  Polymeric NPs (PLGA–PEG, PEG–PCL, etc.) are typically prepared by nanoprecipitation or emulsion solvent evaporation; liposomes and lipid nanoparticles by thin-film hydration or microfluidic mixing; and inorganic cores (gold, iron oxide, silica) by wet-chemical synthesis followed by polymer or lipid coating for biocompatibility and colloidal stability.
2. Targeting ligand conjugation
  Ligands are added by covalent coupling (EDC/NHS amidation, maleimide thiol chemistry, click reactions) or by post-insertion of ligand-PEG-lipids (e.g., Angiopep-2-PEG-DSPE) into pre-formed liposomes. Ligand density must be finely tuned to balance receptor engagement with circulation time; excessive decoration can accelerate clearance or cause receptor saturation without added benefit.
3. Stimuli- and field-responsive elements
  pH-, redox-, or enzyme-cleavable linkers trigger drug release in acidic, GSH-rich, or protease-rich GBM regions. Magnetic (iron oxide) or high-Z (gold, hafnium) cores convert alternating magnetic fields, NIR light, or ionizing radiation into hyperthermia or secondary electrons, enhancing DNA damage, apoptosis, and immunogenic cell death in targeted tumor regions. These biomaterial design principles and the associated surface-modification chemistries are discussed in detail in Nanomedicine: Fundamentals, Synthesis, and Applications and underpin the reproducible, clinically translatable formulations summarized in later sections [25]. Using this modular workflow, ligand-targeted and field-enabled systems have been engineered to improve BBB/BBTB transit, intratumoral accumulation, and therapeutic index in preclinical GBM models.

#### Representative progress in brain-targeted GBM nanomedicines

**Fig. 5:**
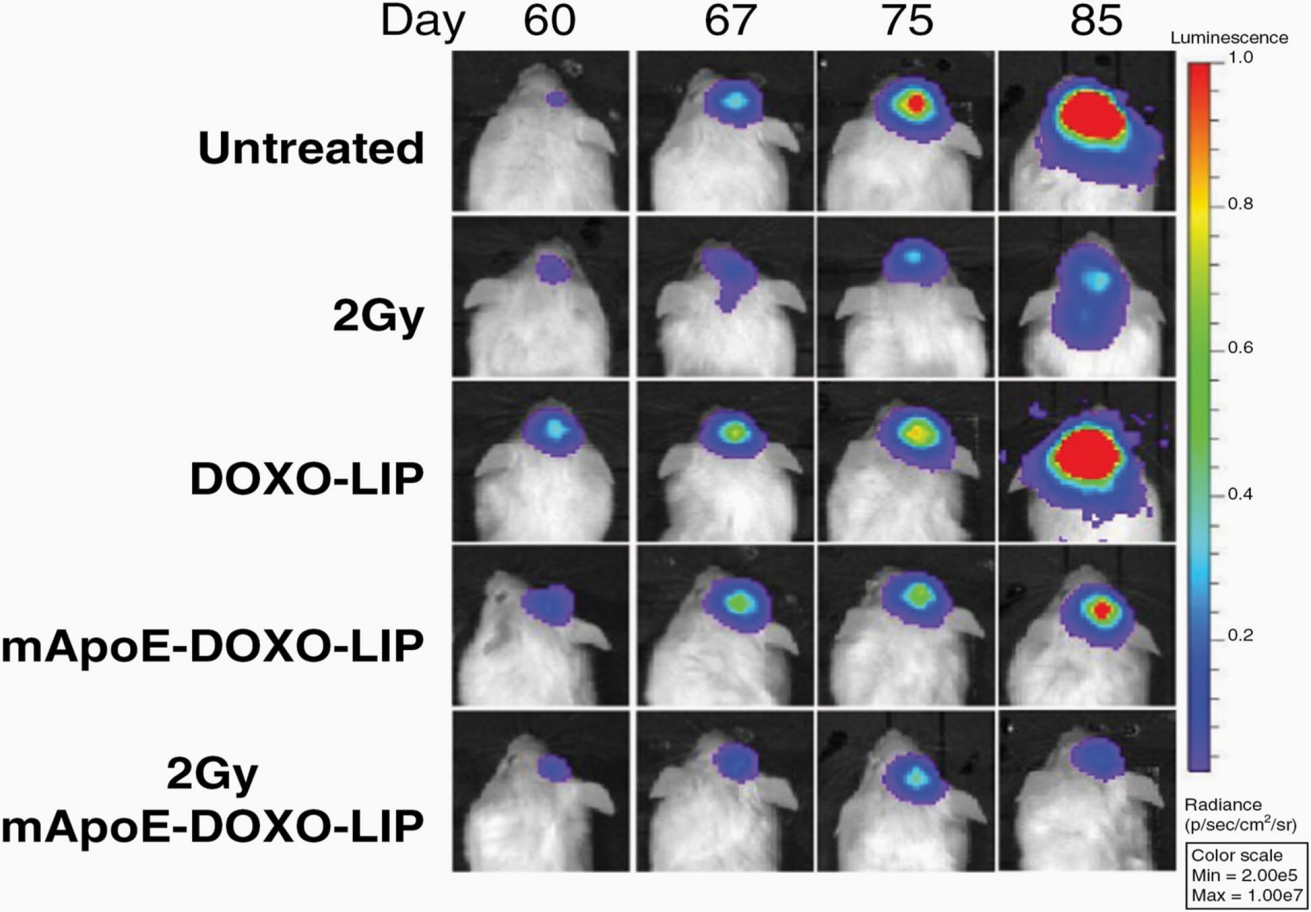
Representative BLI images of tumor–bearing mice untreated (CTR), and treated with untargeted DOXO-LIPs (DOXO-LIP), mApoE-functionalized LIPs as a single agent (mApoE-DOXO-LIP) or concomitant with radiation (2 Gy/mApoE-DOXO-LIP). Reproduced from Pizzocri et al. Neurooncol Adv. 2021, under the CC BY 4.0 licence [77]

Preclinical studies consistently show that actively targeted NPs outperform non-targeted analogues in GBM models. Angiopep-2 functionalized PEG-PCL NPs delivering PTX induce stronger apoptosis and significantly extend survival in orthotopic glioma-bearing mice compared with free PTX or untargeted NPs. Transferrin-modified PLGA NPs co-loading TMZ and radiosensitisers enhanced DNA damage and partially overcame TMZ resistance. cRGD-decorated liposomes and HA-modified NPs improved drug delivery to invasive tumor fronts and GSCs, reducing recurrence in vivo. As another example, mApoE-decorated DOXO-loaded liposomes combined with radiotherapy produced superior tumor growth inhibition and survival benefit compared with non-targeted DOXO-LIPs in orthotopic GBM models (Fig. 5) [77]. Clinically, spherical nucleic acid (SNA) platforms such as NU-0129 show that receptor-guided siRNA-gold conjugates can cross the human BBB and modulate target protein expression inside GBM, providing a mechanistic proof of concept for receptor-guided gene silencing nanotherapies. Representative examples of actively targeted GBM nanomedicines, including those combined with magnetic fields or radiation, are summarized in Table 3.

**Table 3:** Representative brain-targeted nanomedicines for GBM illustrating active targeting modes and anti-GBM mechanisms

Drug	NPs	Targeting type	Animal/cell line	Results	Ref
Chlorotoxin (Ctx) + Ag	Lipophilic Ag entrapped in PLGA-PEG-COOH NPs functionalized with Ctx	Active (GBM cell targeting)	U87MG glioblastoma cells	Ctx-mediated uptake into GBM cells; Ag-induced cytotoxicity (IC₅₀ ≈ 45 μM), enhanced apoptosis vs. non-targeted NPs	[78]
Irinotecan (CPT-11) + shRNA	TCML@CPT-11/shRNA magnetic NPs	Passive + magnetic guidance + AMF	Nude mice, intracranial GBM	AMF-induced hyperthermia + controlled drug release; combination of chemo + gene silencing + magnetic hyperthermia significantly reduces tumor volume and prolongs survival	[79]
Temozolomide (TMZ)	PLGA NPs (TMZ ± NDV)	Active (receptor-mediated)	AMGM5 GBM cells	Improved TMZ uptake; synergistic apoptosis when combined with NDV, suggesting a strategy to overcome TMZ resistance	[80]
Paclitaxel (PTX)	Rg3–liposomes (Rg3–PTX–LPs)	Active (TME/TAM targeting)	C6-bearing rats/mice	Enhanced GBM cell killing; repolarization of tumor-associated macrophages from M2 to M1 phenotype, improving antitumor immunity	[81]
	Angiopep-2-conjugated PEG-PCL NPs	Active (LRP1-mediated BBB/GBM)	Orthotopic glioma-bearing mice	Angiopep-2 enables dual BBB and GBM cell targeting; higher intratumoral PTX, increased apoptosis and significantly improved survival vs. non-targeted NPs	[82]
Doxorubicin (DOX)	Liposomes surface–decorated with mApoE peptide (mApoE–DOXO-LIP) ± radiation	Active (ApoE-mimetic BBB/GBM)	Orthotopic GBM in mice	ApoE-mimetic peptide promotes BBB crossing and GBM uptake; in combination with radiotherapy reduces tumor growth and extends survival compared with non-targeted DOXO-LIP	[77]
	Chitosan-coated magnetic graphene oxide (mGO-CG/DOX) + magnetic field	Active + magnetic hyperthermia	Nude mice xenograft GBM	Magnetic field-induced local hyperthermia plus DOX release achieves strongest tumor inhibition and survival extension vs. DOX alone or non–magnetic controls	[83]

### Stimuli-responsive delivery

In GBM, an ideal nanomedicine should (i) cross the BBB/BBTB, (ii) preferentially accumulate in tumor tissue, and (iii) release or activate its payload predominantly in malignant cells while sparing normal brain. Stimuli-responsive (or “smart”) nanodrug delivery systems (NDDSs) aim to meet these criteria by coupling drug release or activation to specific endogenous biochemical cues (e.g., pH, redox state, enzymes) or exogenous physical fields (e.g., heat, light, magnetic fields, ultrasound). Compared with “always-on” formulations, these systems can sharpen tumor selectivity, enhance anti-GBM activity, and reduce systemic toxicity [84]. Table 4 summarizes representative stimuli-responsive systems investigated for GBM therapy.

**Table 4:** Representative GBM-oriented smart NDDSs and their triggers

Carrier	Type of stimuli	Penetration mechanism	Drug	Drug migration or release rate	References
NPs	MnO_2_	NIR irradiation and LRP (low-density lipoprotein receptor related protein) combination mediated by transcytosis	Temozolomide	Breakdown of HMnO_2_ by H_2_O_2_/GSH in Hollow MnO_2_@PDA(HMP) NPs with release of 90.73 ± 4.34% of TMZ and 95.14 ± 0.52% of Mn^2+^ at 24 h	[85]
NPs	Poly (vinylidene fluoride-trifluoroethylene) [P(VDF-TrFE)] nanoparticles	Receptor-mediated transcytosis via LDL receptor	Nutlin	The migration rate was lower Nut-PNPs + US (25.8 ± 9.1%) than in control cultures (53.8 ± 8.2%)	[86]
Mesoporous silica NPs	Hydrazone-based linker	Passive diffusion	Organoiridium (III) complex	After 48 h, the maximum percentage of release decreased to 60% at pH 6 and 30% at pH 7.4, compared to the maximum release at pH 5	[87]

Broadly, GBM stimuli-responsive systems can be classified into:

(i) exogenous stimuli-responsive systems, triggered by external energy inputs and central to field-enhanced nanomedicine (PTT, PDT, SDT, magnetic hyperthermia, ultrasound-assisted delivery), and.
(ii) endogenous stimuli-responsive systems, which exploit intrinsic GBM features such as acidic pH, high GSH/ROS level, and dysregulated enzymes [88, 89].

#### Exogenous stimuli-responsive delivery

Exogenous stimuli-responsive nanocarriers enable spatiotemporal control of drug release and/or energy deposition at the tumor site by applying external triggers (e.g., mild hyperthermia, NIR light, magnetic fields, ultrasound). For intracranial GBM, clinically relevant triggers include mild hyperthermia, NIR light, magnetic fields, and ultrasound.

1. Temperature-responsive delivery

Temperature-responsive nanocarriers are stable at 37 °C and undergo a phase transition or structural change when heated slightly above this (typically 40 − 45 °C). Above their critical solution temperature, thermosensitive polymers become more hydrophobic, or lipid membranes become more permeable, leading to rapid release of encapsulated drugs. In GBM, such systems are combined with local hyperthermia (e.g., radiofrequency, magnetic, or ultrasound-induced heating) to selectively trigger drug release in the tumor region [90].

For example, thermosensitive polymeric micelles and PLGA-PEG or PLA-PEG-PLA nanoparticles have been evaluated in U87/U251 GBM models for controlled release of temozolomide (TMZ), doxorubicin (DOX), or local anesthetics [84]. These systems show minimal leakage at 37 °C, but accelerated release and enhanced cytotoxicity when exposed to mild hyperthermia, increasing DNA damage and apoptosis in GBM cells while limiting off-target exposure (Table 5) [105, 106]. Liposomal formulations incorporating thermosensitive lipids follow a similar principle and currently represent the most clinically mature thermoresponsive platforms [107].

**Table 5:** Nanoparticles for exogenous stimuli-responsive delivery in GBM

Stimuli	Nanoparticles	Cancer type	Loaded drug	Group/Linker	Ref
Temperature	PLGA-PEG	U-87	TMZ	PLGA-PEG	[91]
	P(MEO2MA-co-OEGMA500)	U251MG	Bupivacaine	OEGMA	[92]
	PLA–PEG–PLA	U-87	DOX	PLA-PEG	[93]
Light	UCNP@CPE-DOX	U87-MG	DOX	Ortho-nitrobenzyl	[94]
	AuNR/PEG-PCL hybrid NPs (cRGD–HNs)	U87MG	DOX	PEG-PCL	[95]
	Amphiphilic spiropyran-functionalized copolymers, poly (isopropylacrylamide-co-spiropyran methacrylate) s	U-87 MG cells	DOX	Spiropyran	[96]
Magnetic	OAMNP-loaded PEG-PLGA NPs	U138 cells	OAMNP	Fe_3_O_4_	[97]
	PEI-PEG-IONPs	U251 cells	Salinomycin	PEI-PEG	[98]
	EDT-IONPs	U251	DOX	EDT	[99]
US	PEG-b-PMBSH	GL261	Sodium borocaptate	Microbubble	[100]
	AP-1-conjugated liposome	GBM8401 cells	DOX	thiol group on each cystine of the AP-1 peptide	[101]
	PEG-PTD-N-PEG	GL261 cells	DOX and Lexiscan	Sensitizers	[102]
			Ce6	Ce6	
Electric	Polypyrrole	GL261	crocin	Polypyrrole polymer	[103]
	Arg-Gly-Asp (RGD) peptide-conjugated gold nanorods (GNRs)	U87MG	GNRs-RGD	GNRs	[104]

2. Light, magnetic, ultrasound-responsive delivery

- Light-responsive nanomedicines

Light-responsive systems for GBM rely predominantly on NIR light, which penetrates deeper and causes less damage than UV or visible light [79]. Upconversion nanoparticles (UCNPs) and gold nanorods (AuNRs) are common cores: UCNPs absorb NIR and emit higher-energy photons that can activate conjugated photosensitizers for photodynamic therapy (PDT), whereas gold nanorods convert NIR into heat for photothermal therapy (PTT) [90]. In U87MG and other GBM models, UCNP-DOX and UCNP photosensitizer systems, as well as cRGD functionalized AuNR/PEG-PCL hybrids, have produced significant tumor regression by combining chemotherapy with ROS-mediated damage or local hyperthermia (summarized in Table 5) [108].

- Magnetic field-responsive nanomedicines

Magnetic nanoparticles (MNPs), typically iron oxide-based, support both imaging and therapy. When incorporated into liposomes, polymeric micelles, or graphene oxide composites, they can be guided toward intracranial tumors by external magnets and heated under alternating magnetic fields (magnetic hyperthermia) [109]. In GBM cell lines (A172, U138, U251, T98), Fe₃O₄ containing nanocarriers loaded with TMZ or DOX achieve substantial cell death when hyperthermia is combined with chemotherapy (Table 5). Moderate heating disrupts DNA repair, enhances drug penetration, and can synergize with radiotherapy; this clinical rationale is exemplified by NanoTherm® in recurrent GBM [90, 110].

- Ultrasound-responsive nanomedicines

**Fig. 6:**
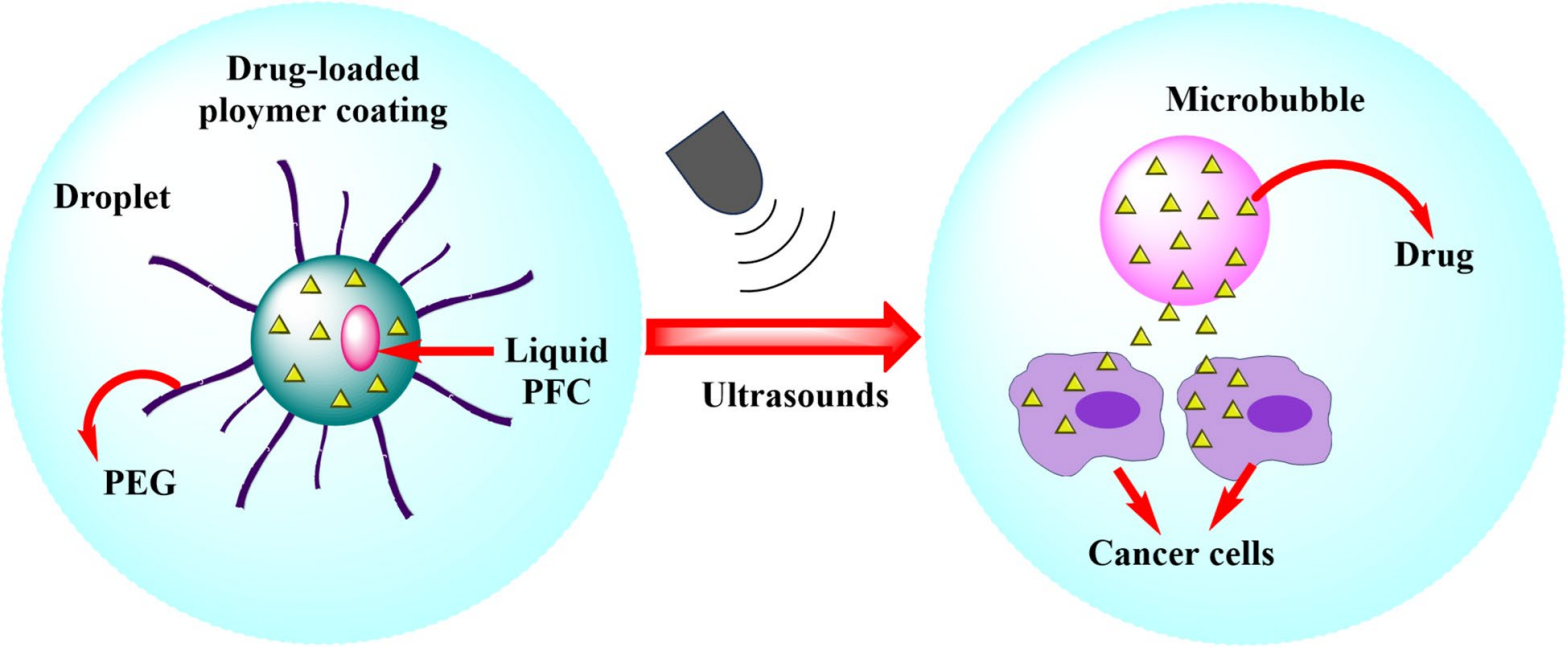
Schematic of a drug-loaded, PEGylated perfluorocarbon (PFC) nanoemulsion droplet that transforms into a microbubble under ultrasound irradiation, leading to local drug release and enhanced uptake by cancer cells [88]

Ultrasound (US) provides mechanical and thermal energy and can both transiently open the BBB and trigger drug release. Ultrasound-sensitive carriers (echogenic liposomes, PFC nanoemulsions (Fig. 6), polymeric aggregates) undergo cavitation-induced disruption or droplet-to-bubble transition within tumor vasculature, releasing encapsulated drugs directly into GBM tissue. In GL261 and related models, ultrasound-triggered systems increase accumulation of DOX, boron agents, or sonosensitizers and enhance tumor control compared to non-triggered delivery (Table 5). Ultrasound-mediated approaches are therefore central to sonodynamic nanotherapy and noninvasive BBB modulation in GBM.

- Electro-responsive systems

Electro-responsive polymers and NPs that release drugs upon application of an electric potential are at a far earlier stage in brain applications. Conductive nanomaterials such as polypyrrole have been explored in GL261 models as proof of concept [111], but technical challenges in safely delivering electric fields to deep brain structures mean that electro-responsive GBM nanomedicine remains a prospective rather than established modality as summarized in Table 5 [112].

#### Endogenous stimuli-responsive delivery

Endogenous stimuli-responsive systems take advantage of GBM-specific biochemical cues to achieve on-site activation. Key triggers include extracellular and endolysosomal pH, intracellular GSH/ROS gradients, and tumor-associated enzymes (e.g., MMPs). These systems can increase the therapeutic window by biasing activation toward tumor tissue and intracellular compartments.

1. pH-responsive systems

GBM exhibits enhanced glycolysis and interstitial acidification; tumor extracellular pH is typically ~ 0.5 units lower than that of surrounding brain, while endosomes/lysosomes are more acidic (pH 5 − 6). pH-responsive nanocarriers exploit this by incorporating acid-labile bonds or protonatable groups that remain intact at pH 7.4 but cleave or rearrange under slightly acidic conditions [113, 114].

Examples include mesoporous silica NPs with hydrazone linkers that release organoiridium complexes more rapidly at pH 5–6, increasing DNA damage in U251 cells. PLGA-PEG NPs conjugated with anti-EGFRvIII antibodies show accelerated DOX release and enhanced cytotoxicity under mildly acidic conditions in U87MG. Dual-drug Apt-Dox-CS-Au-5FU systems exhibit pH-triggered release of DOX and 5-FU and stronger GBM cell killing via cell-cycle arrest [115, 116]. pH-sensitive liposomes retaining calcein at neutral pH but releasing it in acidic GL261 TME [117]. These platforms, summarized in Table 6, illustrate how pH responsiveness can sharpen GBM nanodrug selectivity.

**Table 6:** pH-responsive drug delivery systems in GBM therapy

Carrier	Polymer/Linker/Group	Drug/Marker	GBM model	Findings	Ref
Mesoporous silica nanoparticle (MSN)	Hydrazone-based linker	Organoiridium (III) complex	U251	Compared to physiological pH, the enhanced release of surface functionalized iridium (III) complexes in a mildly acidic environment was demonstrated through pH-responsive delivery of Ir (III) complexes	[118]
PLGA-PEG biocompatible polymeric nanoparticles (NPs) conjugated with anti-EGFRvIII antibody	PLGA-PEG	DOX	U87MG	The in vitro assessment of drug release from conjugated particles revealed a gradual, sustained release of DOX at physiological pH, whereas the initial release was significantly greater in acidic pH conditions	[119]
Apt-Dox-CS-Au-5FU	Chitosan	Fluorouracil and doxorubicin	LN229	The pH-responsive dual drug release induced greater glioblastoma cell death than single drug release through G0/G1 phase cell cycle arrest	[120]
Liposome	Distearoylphospho ethanolamine polyethylene glycol 750 (DSPE-PEG750)	Calcein	GL261	Calcein was released from pH-responsive liposome in acidic TME, while it was retained under physiological conditions	[121]
	distearoylphospho ethanolamine polyethylene glycol 2000 (DSPE-PEG2000)

2. Redox-responsive systems

GBM cells and their TME exhibit elevated GSH and ROS levels relative to normal brain, creating exploitable redox gradients. Redox-responsive nanocarriers incorporate disulfide or diselenide bonds into their backbone, cross-linkers, or drug conjugates; these bonds are stable in mildly oxidizing extracellular conditions but are cleaved by intracellular thiols, triggering payload release.

In C6 and other GBM models, disulfide-crosslinked micelles co-loaded with TMZ and β-lapachone have shown nearly complete payload release under simulated tumor redox conditions, compared with partial release under normal conditions, resulting in synergistic DNA damage and apoptosis. Iron oxide NPs coated with chitosan-PEG and functionalized with O⁶-benzylguanine and chlorotoxin display redox-responsive BG release and markedly enhance TMZ efficacy and survival in orthotopic GBM. ANG-PEG-P(TMC-DTC) based carriers have been used to deliver saporin selectively to U87MG tumors, leveraging reducible linkers for efficient cytosolic delivery [122–128]. Table 7 summarizes representative redox-responsive GBM nanoplatforms and their anti-GBM outcomes.

**Table 7:** Redox-responsive nanocarriers for drug release in GBM

Nanoparticle	Polymer/Linker/Group	GBM model	Drug	Outcomes	Ref
HCA-A2	Cystamine (linker)	C6	Temozolomide and β-lapachone	In vitro, HCA-TMZ-Lapa micelles released 100% of their payload in a simulated tumor microenvironment within 24 h, compared to 43.97% under normal conditions	[13]
Chitosan-PEG (CP) copolymer-coated nanoparticles (NPCP) cross-linked and functionalized with BG (NPCP-BG) and CTX (NPCP-BG-CTX). NPCP consists of a 7.5 nm iron oxide coated with CP	Chitosan-PEG	GBM6 and SF767 cells	Temozolomide (TMZ) and O^6^-benzylguanine	NPs exhibit a redox-responsive drug release profile. In vitro tests confirmed effective release and trafficking of BG within human GBM cells. When combined with TMZ, NPCP-BG-CTX tripled median overall survival compared to untreated animals	[129]
ANG-PEG-P(TMC-DTC)	Poly (ethylene glycol)-b-poly (trimethylene carbonate-co-dithiolane trimethylene carbonate)-b-polyethylenimine	U-87 MG	Saporin	Effective tumor inhibition and significantly improved survival rate	[130]

3. Enzyme-responsive systems

Multiple enzymes, including MMP-2/9, cathepsins, and glycosidases, are dysregulated in GBM. Enzyme-responsive nanocarriers incorporate cleavable peptide or sugar linkers that are selectively degraded by these enzymes, enabling tumor–specific activation or release [131]. In 9L and C6 glioma models, enzyme cleavable coatings on magnetic or gold NPs have achieved MMP-dependent activation, enhancing tumor retention and therapeutic efficacy. CXCR4-targeted AMD3100-PLGA NPs provide another example: enzymatic degradation of the polymer matrix in the tumor environment enables sustained release of the CXCR4 antagonist, disrupting chemokine signaling and limiting GBM cell migration. While still earlier in development than pH- or redox-responsive platforms, enzyme-responsive systems offer a route to match nanodrug activation to specific protease signatures within the GBM TME as shown in Table 8 [135].

**Table 8:** Enzyme-responsive nanoparticles

Carrier	Cell lines/GBM/brain tumor model	Enzyme trigger target	Enzyme activity/Survival (day)	Drug delivery type	Cargo/design principle	Key anti-GBM outcome	Ref
PEG-β-Glu-MNP	9L-glioma	β-Glucosidase was conjugated onto nanoparticles to retain its enzyme activity on the nanoparticles	65.4%	In vitro/In vivo	β-glucosidase was conjugated on aminated magnetic iron oxide nanoparticles using the glutaraldehyde method (β-Glu-MNP), and further PEGylated via N-hydroxysuccinimide chemistry to enhance plasma stability	Conjugated β-glucosidase showed 73.0% activity for β-Glu–MNP and 65.4% for PEG-β-Glu-MNP. PEG-β-Glu-MNP accumulated well in tumor tissue, with an iron content of 627 ± 45 nmol Fe/g and β-glucosidase activity of 32.2 ± 8.0 mU/g	[132]
AuNP-AK-R AuNP-CABT-R	C6 GBM-bearing mice	Legumain overexpressed in GBM	56%	In vitro/In vivo	AuNP-AK-R and AuNP-CABT-R aggregate in the presence of legumain and for doxorubicin (DOX)-loaded chemotherapeutic effect	AuNP-A&C-R actively targeted the integrin αvβ3 receptor on blood–brain barrier (BBB), mediated transcytosis of particles across the BBB, and then targeted the receptor on the GBM cells. Also, improved chemotherapeutic effect to C6 GBM-bearing mice	[133]
AMD3100-PLGA	U87 cells	Esterase	57%	In vitro/In Vivo	Tandutinib-based esterase-responsive prodrugs and AMD3100-modified PLGA NPs to penetrate the BBB and determination of the optimal PLGA NP size	Overcome the BBB Responds to the GBM microenvironment Inhibited tumor growth and effectively prolonged the survival of tumor-bearing mice	[134]

### Multi-responsive systems

Multi-responsive NDDSs integrate two or more orthogonal triggers (e.g., pH/redox/enzyme cues with NIR light, ultrasound, magnetic fields, or mild hyperthermia) to increase spatiotemporal precision and reduce off-target activation in GBM [136]. Han et al. engineered block copolymer micelles bearing both photo-responsive ONB and redox-responsive disulfide groups; rapid release occurred in response to UV light and slower release upon addition of a reducing agent, generating a combined fast/slow release profile desirable for staged drug-delivery applications [137]. Collectively, these multi-stimuli platforms exemplify how integrating endogenous and exogenous triggers can sharpen the therapeutic window of GBM nanomedicines, enhance the efficacy of field-based therapies, and potentially mitigate intratumoral heterogeneity by activating treatment only in regions that simultaneously exhibit specific biochemical and physical conditions.

## Nanomedicine platforms for GBM

### Organic nanocarriers: lipid-based and polymeric systems

#### Lipid-based nanoparticles

Lipid-based nanoparticles (liposomes, solid lipid NPs, lipid nanocapsules) are among the most biocompatible nanoparticles that are particularly effective in the delivery of hydrophobic drugs. They have the capability of encapsulating agents such as paclitaxel, doxorubicin, carmustine, with aqueous solubility enhanced by several orders of magnitude.

**Fig. 7:**
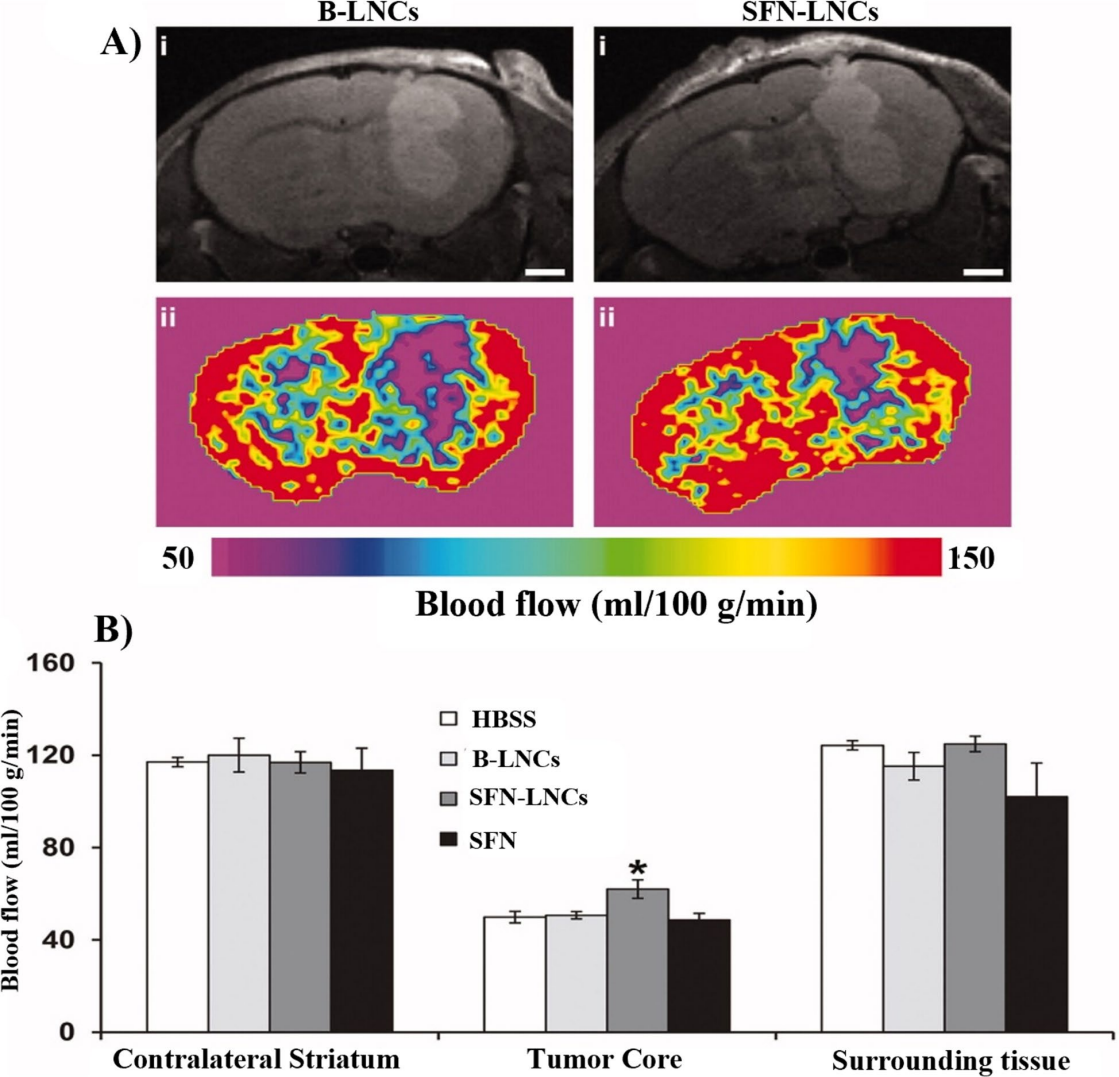
**A** Perfusion MRI images of U87MG glioma-bearing mice treated with B-LNC and SFN-LNC (scale bar = 1 mm). The top panels (i) display T2-weighted morphological images, while the bottom panels (ii) show perfusion maps. **B** Graph illustrating blood–flow values in the tumor core, surrounding tissue, and contralateral striatum Reproduced from Clavreul et al. [143]. Drug Delivery 25 (2018) 1756–1765, an open-access article distributed under the terms of the Creative Commons CC BY license

PEGylation of lipids increases circulation time and surface ligands (e.g., transferrin, Angiopep–2) facilitate receptor-mediated transcytosis across the BBB. Examples of lipid-based GBM nanocarriers (e.g., CpG-augmented SLNs, HDL-like particles) as lists in Table 9 and their effects in vivo (Fig. 7) Ligand-targeted liposomes, e.g., HA-functionalized liposomes are much more effective at targeting glioma [143].

**Table 9:** Lipid-based nanoparticles used in drug delivery systems for glioblastoma

**Nanocarrier**	**Drug**	**Delivery route/Targeted pathway**	**GB model**	**TEM/Zeta potential**	**Results**	**Ref**
CS-coated LNC	Paclitaxel CpG	CED	GL261-glioma-bearing mice	+ 25 mV	Survival: PTX-loaded LNC + CpG = 31 days	[138]
Lid-FA-Lip	Lidocaine	PI3K/AKT	U87 cells	–8.65 ± 0.77 mV	80% of the lidocaine is released in 10 h. Lid-FA-Lip exhibited a slightly stronger antiproliferative effect	[139]
Ionizable lipid Dlin-MC3-DMA DSPE-PEG-NH2	siRNAs encoding PLK1	CED	U87MG	− 8.2 ± 0.7 mV, 3.8 ± 1 mV	Survival: HA-LNPs-siLuc: 34.5 days HA-LNPs-siPLK1: N/A—60% of animals survived the study period of 95 days	[140]
sHDL DMPC POPC	docetaxel CpG	CED + RT	Mouse GL26 cells	~ 10 nm	Survival: DTX-sHDL-CpG + RT: median survival not reached	[141]
DMPC: DOTAP: Chol: DiD	–	IAD	C6 glioma cells	− 9.53 mV	Large and cationic liposomes display better accumulation in GBM	[142]

**Fig. 8:**
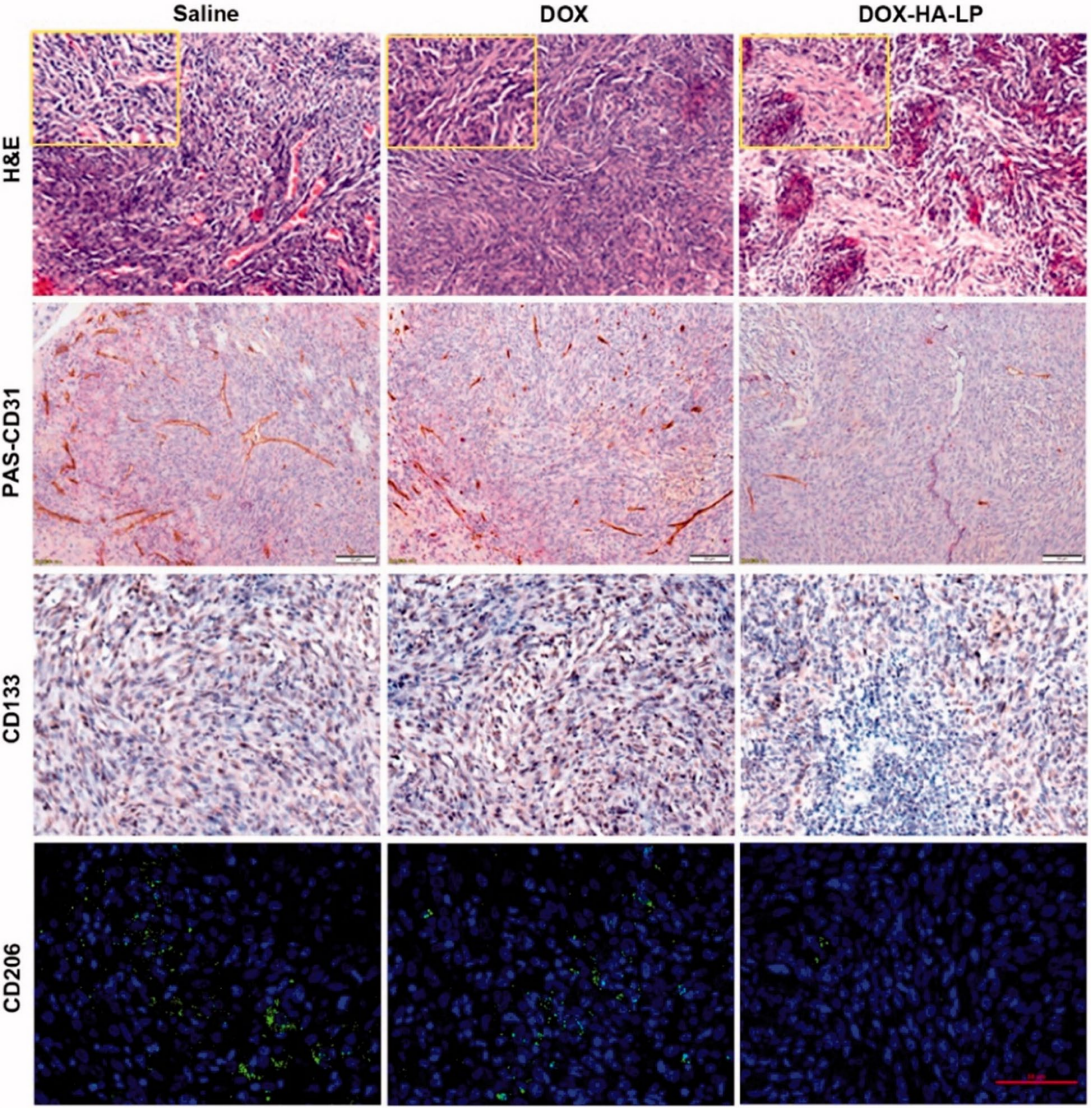
Shows H&E staining of the brain glioma site 24 h after administering saline, free DOX, and DOX-HA-LPs. Scale bars are 50 µm and 20 µm [144]. Reproduced from Yang et al. [144], open-access article distributed under the terms of the Creative Commons Attribution (CC BY) licence

According to Yang et al., the DOX-HA-liposomes (~ 156 nm) were much more effective in killing C6 glioma cells than the non-targeted DOX liposomes (Fig. 8) [144]. All of these studies demonstrate the ways in which ligand-functionalized LBNPs can improve GBM-cell uptake, intratumoral retention, and cytotoxicity.

#### Polymeric nanoparticles

**Fig. 9:**
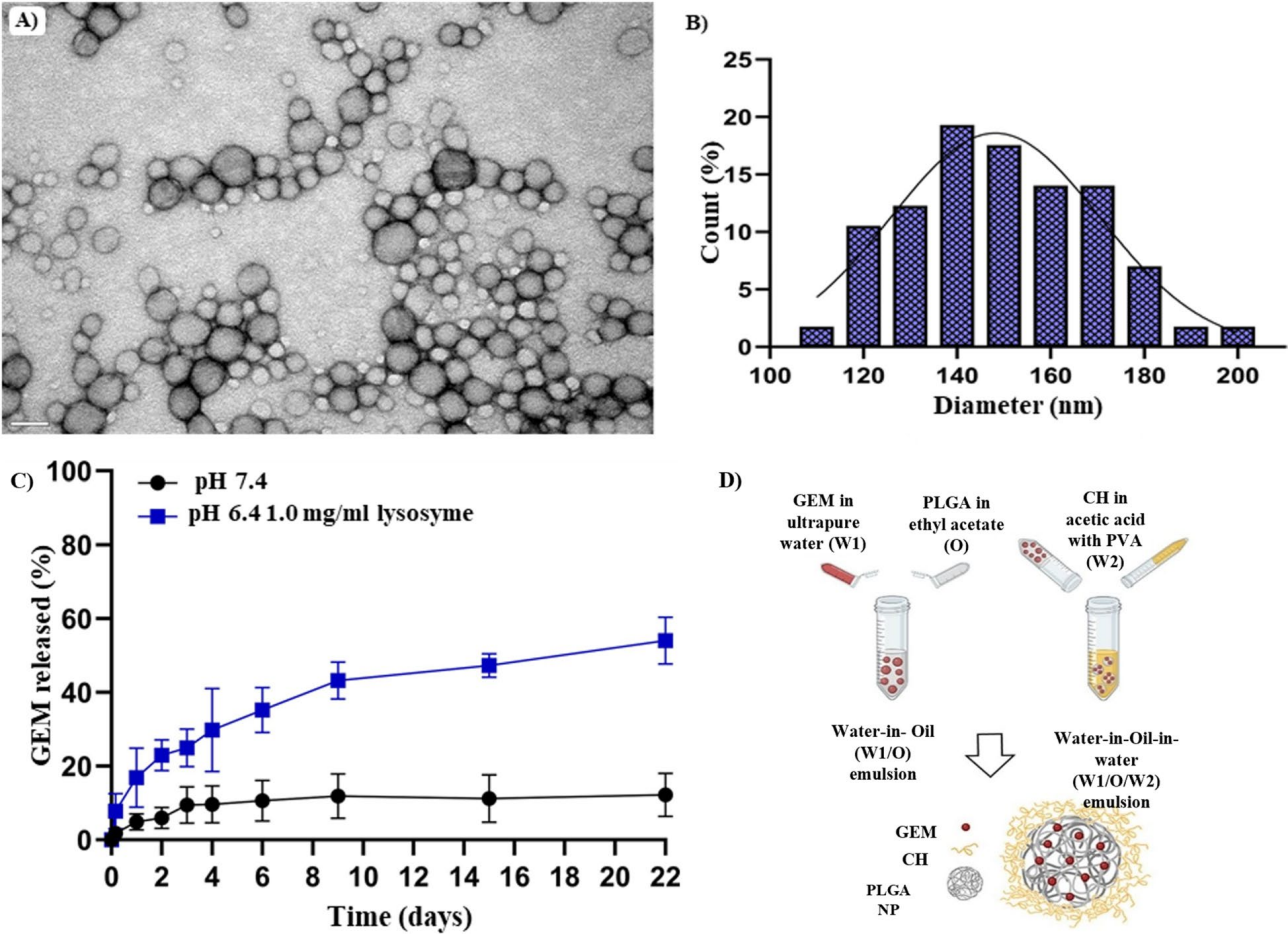
**A** TEM image and **B** histogram of the prepared GEM-loaded CH-PLGA NPs **C** in vitro release profile GEM-loaded CH-PLGA NPs and **D** GEM-loaded CH-PLGA NPs preparation method. Reproduced unmodified from Ramalho et al. [152], open access under a Creative Commons license

Polymeric NPs (PLGA, PEG-PLA, polyacrylates, etc.) allow controlled, sustained release, and may entrap small drugs, as well as nucleic acids (Table 10) [150]. Critically, particles < 100–200 nm penetrate disrupted BBTB more effectively, while PEGylation reduces opsonization [151]. For example, Ramalho et al. made ~ 150 nm chitosan-coated PLGA NPs carrying gemcitabine (GEM) for nasal delivery; these NPs showed enzyme-responsive release and sensitized TMZ-resistant GBM cells (T98G) to TMZ (Fig. 9A–D) [152]. As shown in Fig. 9, these ~ 150 nm particles exhibit enzyme-responsive GEM release, and their double-emulsion preparation method is summarized in panel D [153]. Combination treatment with CH-GEM NPs and TMZ increased TMZ activity 7.5-fold in TMZ-resistant T98G cells, indicating strong anti-GBM combination [154].

**Table 10:** Drug delivery across the blood–brain barrier by polymeric nanoparticles

Nanocarrier	Drug	Glioblastoma model	Size	Methods of crossing BBB	Ref
PEG-PLGA/NPs	DOX	U251 cells	24.11 ± 1.36	Modified by I_6_ P_8_ peptide	[145]
RGDyC-Mpeg-PAMAM	Arsenic trioxide (ATO, As2O3)	C6 cells	21.60 ± 6.81 nm	Modified with RGDyC	[146]
PLGA-PEG	PTX	Rats bearing 9L gliosarcomas	69 ± 4 nm	PS	[147]
DSPE-PEG_2000_-COOH	DOX	C6 glioma cells	167.2 ± 1.9	Angiopep-2 peptide and AS1411 aptamer	[148]
mPEG-b-p(HPMAm-Bz) micelles	VAL + PAN	Mouse GL261	65 nm	Combine with MB and US	[149]

*PS* pH-sensitive, *MB + US* microbubble plus ultrasound-mediated delivery

Polymeric NPs can be easily functionalized with targeting ligands [155]. PEG-modified NPs are more efficient in their uptake and cytotoxicity in relation to U87MG compared to non-PEG formulations [156]. Sahoo et al. designed angiopep-2-grafted PAMAM dendrimers (with or without PEG) for LRP1-mediated TMZ delivery; the PEGylated, Angiopep-2–targeted system (TMZ@Den-PEG₂-ANG) produced the greatest cytotoxicity in U87MG cells (IC50 106.62 ± 11.43 µM at 24 h; 85.90 ± 9.12 µM at 48 h) [156], illustrating how dual PEGylation/ligand targeting strengthens anti-GBM activity.

**Fig. 10:**
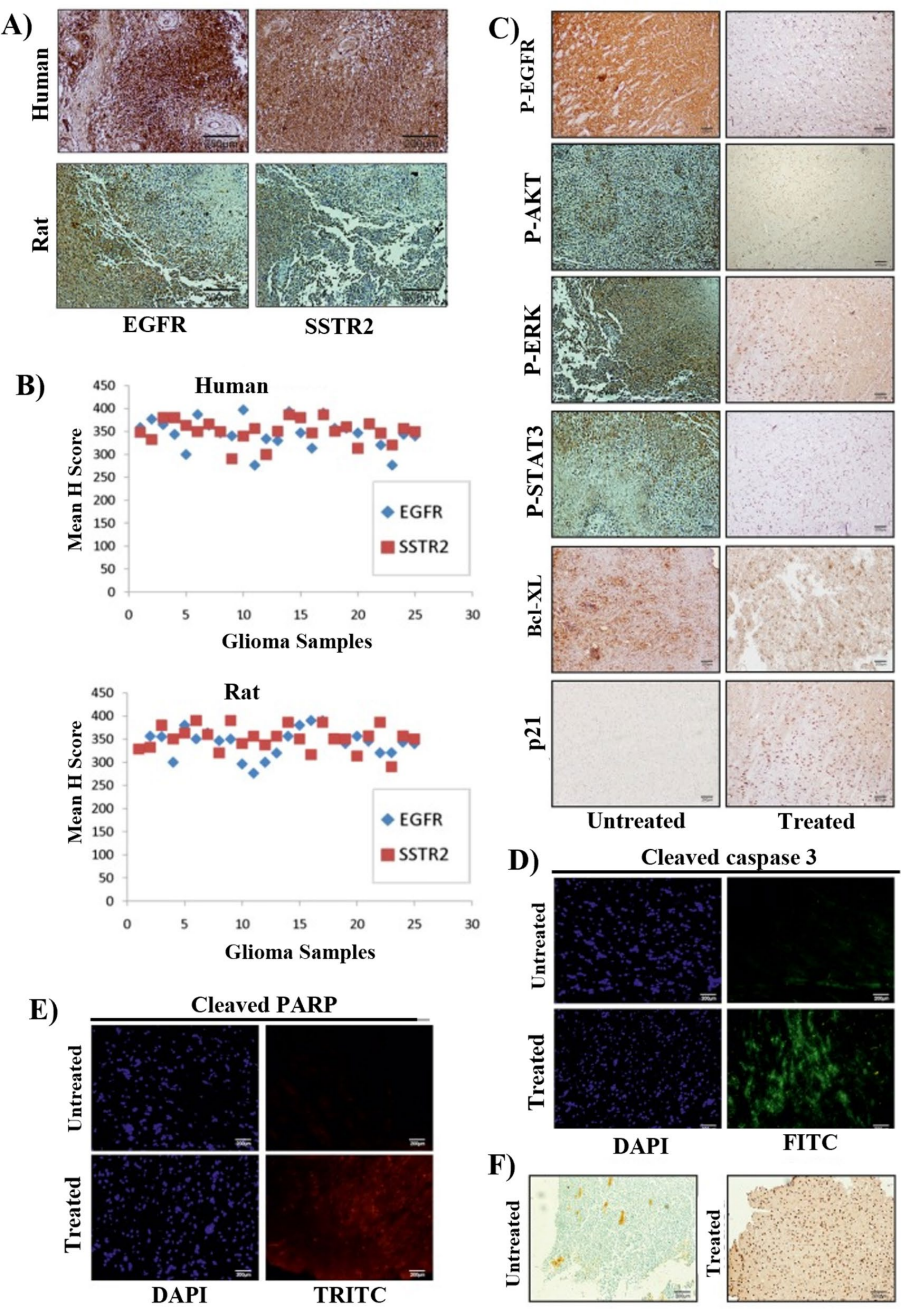
Inhibition of the EGFR pathway in vivo by SSTR2pep-DIM-NP [157]. Reproduced from Bhowmik et al. [157], Oncotarget (2017), under the Creative Commons Attribution 3.0 licence (CC BY 3.0)

Bhowmik et al. used brain-penetrating PLGA NPs loaded with 3,3′-diindolylmethane (DIM) and decorated with an SSTR2-targeting peptide. In human and rat glioma samples, SSTR2 and EGFR were co-expressed; peptide-tagged DIM NPs suppressed EGFR downstream signaling (p-EGFR, p-AKT, p-ERK, p-STAT_3_), reduced Bcl-XL, increased p21, and induced apoptosis by caspase-3/PARP activation and TUNEL positivity in vivo (Fig. 10) [157].

**Fig. 11:**
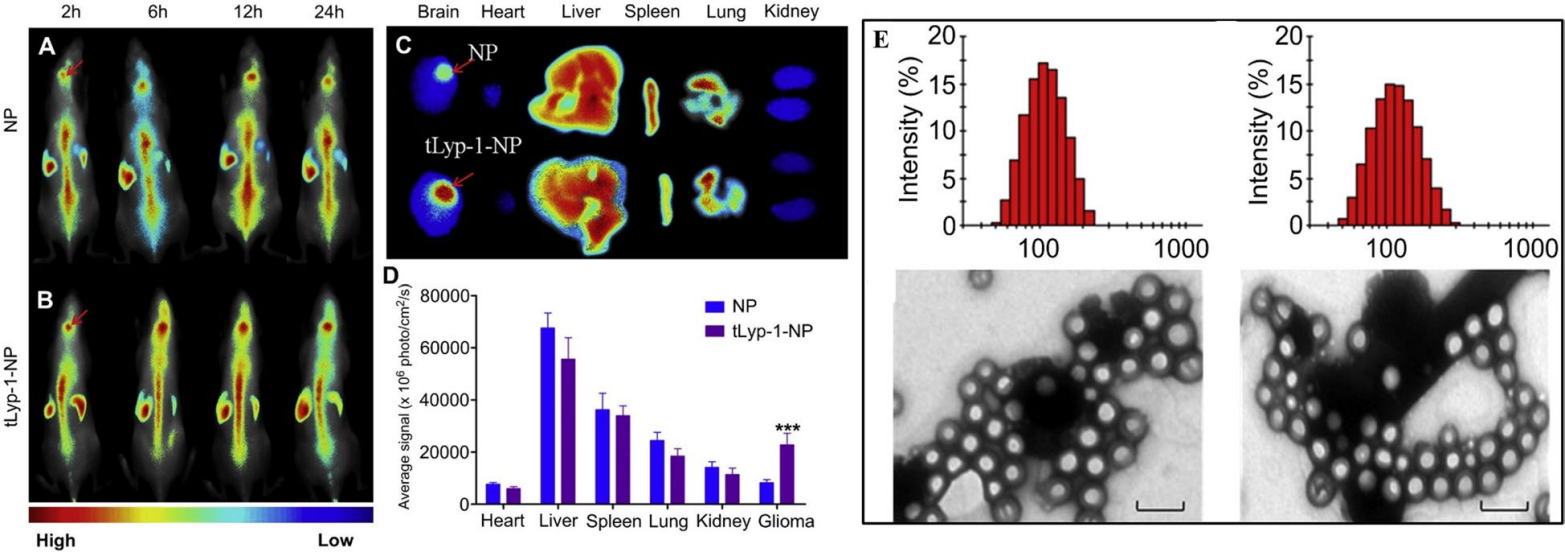
In vivo near-infrared imaging of mice with intracranial C6 glioma, intravenously treated with 200 mL of DiR-labelled NPs (**A**) and tLyp-1-NPs (**B**) via the tail vein at 2, 6, 12, and 24 h. It includes in vivo fluorescence imaging of dissected organs 24 h post-injection (**C**), along with semiquantitative analysis of fluorescent intensities in various organs and the glioma (**D**). The red arrows indicate the tumor site. **E** Presents characterization data for NPs and tLyp-1-NPs, including particle size distributions and TEM images [158]. Reproduced from Hu et al., Biomaterials 34 (2013) 620–632, with permission from Elsevier [158]

Another example is Hu et al.: tLyp-1 peptide-PEG-PLA NPs loaded with paclitaxel (≈ 111 nm) showed maximal C6 glioma accumulation ~ 12 h post-injection, outperforming non-targeted NPs (Fig. 11) [158]. This confirms dual passive (EPR) and peptide-mediated targeting.

Dendrimer-based systems further extend mechanistic possibilities: Pep-PEG-PAMAM dendrimers exploit IL-13Rα2 for BBB/BBTB crossing and GBM targeting [159].

Miklaszewska et al. used amphiphilic dendrimers that enable microglia-specific siRNA delivery without loss of viability and SRL-decorated PAMAM-PEG systems achieved high in vitro gene-transfection efficiency via LRP, albeit with potential competition from endogenous lactoferrin [160].

### Inorganic nanoparticles

Inorganic nanoparticles including gold, silver, graphene-based materials, silica, hydroxyapatite, iron oxide, and related ceramics provide high atomic numbers, magnetic responsiveness, and strong photothermal/photodynamic properties that are advantageous for GBM diagnosis and therapy [35]. Metallic nanoparticles (Au, Ag, Fe) exhibit tunable optical and magnetic characteristics, making them potent radiosensitisers and physical-field transducers [161]. In radiotherapy, high-Z nanomaterials (e.g., Au) enhance local dose via increased photon attenuation and secondary electron emission; in PTT/PDT, Au nanostructures efficiently convert NIR light into heat or ROS, triggering apoptosis, necrosis, and vascular damage in GBM tissue [162, 163].

Combining metal nanoparticles with radiotherapy and immunotherapy is an emerging strategy. Gold-core silica-shell nanoparticles (Au@SiO₂) plus low-dose X-irradiation augmented PD-L1 blockade in GL261 brain tumor models, promoting immunogenic cell death and stronger anti-tumor immune responses [164]. Superparamagnetic iron oxide nanoparticles (SPIONs) serve as contrast agents, drug carriers, and hyperthermia mediators. The NanoTherm® ferrofluid (aminosilane-coated SPIONs ~ 15 nm) is injected into recurrent GBM and activated by an alternating magnetic field to generate local hyperthermia, selectively damaging tumor cells and acting as a radiosensitizer [165]. Coating MNPs with collagen, chitosan, or gold can further improve biocompatibility, targeting and multimodal imaging [166, 167]. Representative inorganic platforms are listed in Table 11, including silica-based chemo-sonodynamic systems combined with focused ultrasound (FUS), Au@SiO₂ for radio-immunotherapy, and transferrin-modified SrBi₂Ta₂O₉ particles as FUS-enhanced radiosensitizers.

**Table 11:** Inorganic nanoparticle formulations for GBM

Nanocarrier	Drug dose	Delivery route	Glioblastoma model	Survival	Ref
SiO_2_ BTDS Cu_2_ -xSe-PEG	DOX 5 mg/kg	CED + FUS	U87MG mouse model	DOX-HCu/FUS: 52 days	[168]
Au@SiO_2_	Atezolizumab Low-dose	X–ray irradiation	GL261	40% survival rate	[164]
SrBi_2_Ta_2_O_9_ DSPE-PEG 2000-Transferrin	SBTO nanoparticles (3 mg/kg)	CED + FUS	U87MG mouse model	SBTO nanoparticles + FUS: 67%	[169]
Ang-Fe-Au	–	Magnetic field-induced hyperthermia	C6 glioma cells	Increased survival time by 7 days	[170]

Key: PS denotes pH–sensitive, MB+US denotes microbubble plus ultrasound–mediated delivery

**Fig. 12:**
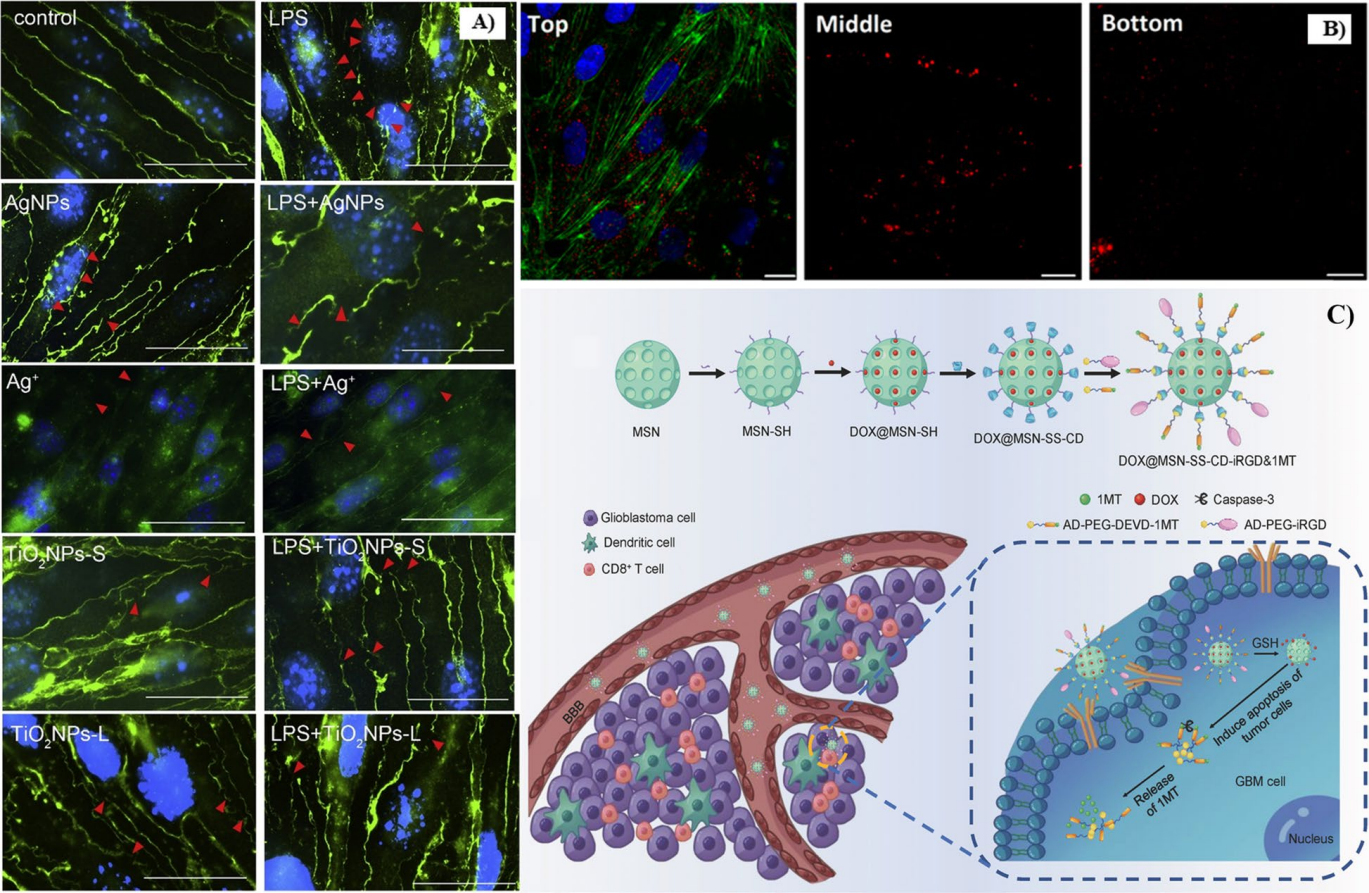
**A** Ag +, Ag NPs, and TiO_2_ NPs within the BBB model. **B** Confocal images of the in vitro BBB exposed to PSi-Lf NPs. **C** Mechanism of the combined chemo–immunotherapeutic mesoporous silica nanoparticles [171–173]

Silver and titanium dioxide nanoparticles can traverse in vitro BBB models, highlighting that inorganic NPs can be engineered for brain access (Fig. 12A) [171]. PEGylated, lactoferrin-modified porous silicon NPs (~ 25 nm) achieved fourfold higher BBB transport efficiency than unmodified particles (Fig. 12B) [172]. Mesoporous silica nanoparticles (MSNs), with large internal surface area and tunable pores, have been used to co-load chemotherapeutics and immune adjuvants, enabling combined chemo-immunotherapy and TME modulation in glioma models (Fig. 12C) [173].

#### Hydrogel-based nanoparticles

Hydrogels provide an attractive platform for local, postoperative GBM therapy, particularly when combined with nanoparticle formulations (Table 12). Their ability to form in situ depots within the resection cavity enables sustained release of chemotherapeutics, immunomodulators, and photosensitizers, limiting systemic exposure and delaying recurrence [178]. Incorporation of nanoparticles allows fine control of release kinetics and integration of targeting ligands, enzyme-responsive motifs, and photodynamic or chemodynamic functions [179, 180]. Hydrogels based on drug-loaded lipid nanocapsules (GemC12-LNC, PTX, DOX, salinomycin, curcumin) have shown good brain tolerability and prolonged survival across U251, T98G, 9L-LacZ, and U87MG models [181–183]. Gazaille et al. further decorated GemC12-LNC hydrogels with neurofilament peptide NFL-TBS.40–63 (NFL); in a GBM resection model, GEM-NFL-LNC hydrogels targeted residual tumor cells and extended median survival to 74 days versus 38 days (untreated) and 44 days (non-targeted GEM-LNC) [184]. Zhao et al. developed an injectable MMP-responsive triglycerol monostearate (Tm) hydrogel co-loaded with TMZ and O⁶-benzylamine (BG) [185]. Elevated postoperative MMP levels triggered in situ drug release in TMZ-resistant C6 models, increasing survival to 54.5 days versus 39.5 days with free TMZ.

**Table 12:** Hydrogels are used for postoperative glioblastoma treatment

**Nanocarrier**	**Drug**	**Deliveryroute**	**GBM model**	**Monitoring/Analysis**	**Outcomes**	**Ref**
PEG750-p(CL-co-TMC) PEG–DMA	TMZ	In situ implantation	U87MG	RTD	Sustained release of TMZ over 1 week Good tolerability in brain tissue Most importantly, it showed potent in vivo antitumor efficacy	[174]
BSA CMC-g-PNIPAAmMA DTPA_Gd_/bPEI	EPI + PTX	In situ implantation	U87MG and MBR614	MRI	The average survival of gliosarcoma-bearing (MBR 614 or U87) mice receiving BSA/PTX NPs incorporated hydrogel Gd/EPI increased to 63 days or 69 days, with no tumor recurrence observed	[175]
PEO-PPO-PEO PLGA-PEG-PLGA	Salinomycin	Intratumoral administration/Injection	U251	Quantitative RT-PCR	Animal studies using subcutaneous U251 xenografted nude mice showed that the Pluronic + salinomycin hydrogel decreased tumor growth significantly more than free salinomycin and PBS treatments by fourfold and sixfold, respectively within 12 days	[176]
MPDA M1NVs Fibrinogen THR	DOX	In situ implantation	GL261 model	PDT	Improved the immune microenvironment This hydrogel system, when combined with a near–infrared laser, effectively encouraged ongoing T cell infiltration, restored T cell effector activity, reduced the infiltration of myeloid-derived suppressor cells and regulatory T cells, and thus demonstrated a potent antitumor immune response, leading to significant tumor growth suppression	[177]

A multifunctional CP&CL@RNPPTX-Gel hybrid, embedding self-illuminating chlorin e6-luminol conjugates, glioma-targeted PTX prodrug NPs, and copper peroxide nanodots, achieved combined chemo, chemodynamic, and photodynamic therapy with strong local tumor control and immune activation in a postoperative U87MG model [186]. Wang et al. reported an iRGD- and anti-CD47–containing PTX hydrogel (αCD47-PTX-RGD) that filled the resection cavity, promoted macrophage-mediated phagocytosis and T cell responses, delayed recurrence and improved survival versus PTX-only hydrogels [187].

### Biomimetic nanoparticles

Biomimetic nanoparticles (BMNPs) harness natural biological components such as cell membranes, lipoproteins, or virus-like shells to evade immune clearance, prolong circulation, enhance BBB/BBTB transport, and achieve tumor-specific targeting. By retaining native membrane proteins and ligands, BMNPs engage physiological trafficking and homotypic recognition pathways, which is particularly valuable for infiltrative GBM [167, 188].

#### Cell membrane–coated NPs for immune evasion

Cell membrane–coated NPs derived from macrophages, neutrophils, dendritic cells, erythrocytes, or tumor cells can inherit extended circulation, antigen recognition and immune modulation from their source cells [188]. GBM cell membrane coating further enables homotypic adhesion and deep penetration into invasive margins [189].

These coatings also allow BMNPs to exploit homotypic adhesion, facilitating deep penetration into infiltrative GBM margins and residual cells after surgery. This section reviews cell membrane–driven BMNPs (Table 13) and other biomimetic variants [194].

**Table 13:** Cell membrane–driven BMNPs for GBM treatment

Nanoparticles	Therapeutic agent	Dose	GBM model	Zeta potential	Effect	Ref
IL-12 mRNA@cRGD-CM-CaCO_3_ NPs	IL-12 mRNA	0.25 mg/kg	GL261 cells	3.5 mV	Nanoparticles cross the BBB and specifically target tumors via homing and homotypic recognition. This approach offers a platform for ultrasound-immune synergistic therapy of brain tumors	[62]
UCNPs@Ce6/3HBQ@CM	3HBQ; Ce6	75 mg/kg	U87MG	− 32.9 ± 1.0 mV	The nanocomposite can target GBM and accumulate in the tumor site	[190]
CM@AIE NPs	AIEgens	200 μg/mL	U87-MG	7 mV	Efficient transfer into primary human T cells enhanced their affinity for GBM cells	[191]
Virus-mimicking membrane-coated nucleic acid nanogel Vir-Gel embedded with therapeutic miRNA	miR155	2 mg/kg	C57/BL6 mice bearing glioma	− 18 mV	Specifically targeting M2–microglial and macrophage cells, promoting the fusion of endosomal membrane and erythrocyte membrane, and increasing the release of nanogel in the cells	[192]
PM-HDOX	Doxorubicin	2.5 mg/kg	U87 cells, U251 cells	28.9 ± 0.8 mV	The inherent ability to cross the compromised BBB and target the surgical margin effectively inhibits the growth of residual tumors after surgery, extending the median survival time of mice	[193]

Du et al. constructed MnO₂-DOX-C6 NPs coated with C6 glioma membranes. The biomimetic shell conferred homotypic targeting and pH-responsive release; ~ 66.8% of DOX was released at pH 5.0 within 48 h. In vitro and in vivo, MnO₂-DOX-C6 induced apoptosis and significantly suppressed C6 tumor growth [195]. Han et al. coated PEI25k/pDNA complexes with C6 membranes to generate negatively charged, stable gene-delivery NPs. CM-coated PEI25k/pHSVtk complexes displayed enhanced homotypic transfection and superior tumor regression compared with non-coated controls [196].

Ma et al. developed a DCM@PLGA/RAPA, rapamycin-loaded PLGA NPs cloaked with activated dendritic cell membranes. This platform crossed the BBB, promoted dendritic-cell maturation, activated CD8⁺ T cells and NK cells, and inhibited orthotopic glioma growth while inducing protective antitumor immunity [197]. Other BMNPs encapsulate acoustic sensitizers or photosensitizers within cancer cell membranes, enabling NIR- or ultrasound-triggered PDT/SDT with precise GBM targeting, improved tumor control, and limited systemic toxicity [198, 199].

Researchers are exploring biomimetic drug delivery systems integrated with native cell membranes for cancer treatment through light therapy. Sun’s et al. utilized a red blood cell (RBC)-based approach focused on targeting tumor angiogenesis. The modified RBCs were loaded with the anticancer drug DOX and photothermal agents composed of indocyanine green associated with bovine serum albumin nanocomplexes. The IB&D@RBC–RGD significantly enhanced the release of these agents and boosted cytotoxic effects against U87MG and 4T1 cells due to the deep penetration capabilities of near-infrared (NIR) light [200].

#### Lipoprotein–mimetic nanocarriers for BBB/BBTB transport

Kadiyala et al. developed a chemo-immunotherapy approach using synthetic HDL (sHDL) nanodiscs that are loaded with CpG, a Toll-like receptor 9 agonist, and docetaxel (DTX), targeting GBM effectively. The introduction of DTX-sHDL-CpG nanodiscs into tumors resulted in tumor shrinkage and stimulated antitumor CD8^+^ T cell responses within the brain tumor microenvironment, without significant off-target effects. When DTX-sHDL-CpG treatment was combined with radiation therapy, it led to tumor reduction and achieved long-term survival in 80% of the treated mice, who also remained tumor-free after being re-challenged. This indicates the establishment of immunological memory against GBM [141].

Bertrand et al. administered ANG1005 and Cy5.5Angiopep-2, which are targeted therapies, to mice with orthotopic glioma tumors and found a correlation with high expression of LRP1. They discovered that reducing LRP1 activity through RNA silencing or by using competitors decreased the uptake of ANG1005 and Angiopep-2 in U87 glioblastoma cells. In contrast, LRP1 expression and the endocytosis of these agents increased in U87 cells when exposed to hypoxic and acidic conditions, which simulate the aggressive tumor microenvironment [201].

## Clinical translation of nanomedicines for GBM treatment

Although most GBM nanomedicines remain preclinical, several nano-enabled, often field activated, therapies have reached early-phase clinical evaluation.

- Magnetic hyperthermia with SPIONs (NanoTherm®)

NanoTherm® is an aqueous suspension of aminosilane-coated SPIONs (~ 15 nm) administered intratumorally or into the resection cavity in recurrent GBM [202]. Under alternating magnetic fields, the particles generate local hyperthermia, leading to direct tumor cell death and radiosensitization. Single-arm trials reported median overall survival of around 13–23 months from first diagnosis when NanoTherm® was combined with RT, exceeding historical RT-alone controls [203]. NanoTherm® holds a CE mark in Europe for recurrent GBM, and ongoing studies (e.g., ANCHOR/ANCHIALE, NCT06271421) are reassessing its adjuvant potential [204].

- Spherical nucleic acid nanoconjugates (NU–0129)

NU–0129 is a gold–core spherical nucleic acid (SNA) carrying siRNA against BCL2L12. A first-in-human phase 0 trial in recurrent GBM showed that systemically administered NU-0129 crossed the BBB/BBTB, accumulated in tumor tissue and reduced BCL2L12 expression in resected samples, providing proof-of-concept for RNAi-based SNA therapy in human GBM [205].

- Liposomal and polymeric nanocarriers

CED-delivered nanoliposomal irinotecan (CPT–11) has shown acceptable safety and MRI-verified local distribution in recurrent high-grade glioma, though efficacy has been modest to date. Glutathione-PEGylated liposomal DOX (2B3–101) demonstrated BBB penetration and brain-tumor uptake with manageable toxicity but limited objective responses in early-phase trials. SGT-53, a transferrin-targeted cationic liposome encoding wild-type p53, confirmed the feasibility of systemic p53 gene delivery and p53 expression in GBM biopsies when combined with TMZ, even though its phase II study ended early for non-safety reasons.

- Other nano-enabled radiosensitizers and oxygen carriers

Hafnium-oxide nanoparticles (NBTXR_3_) have enhanced RT responses in soft-tissue sarcoma and head-and-neck cancers and are being explored in brain tumors, illustrating the potential of high-Z dose-enhancement strategies. Perfluorocarbon-based oxygen nanodroplets (e.g., Nano TMZ) are also under investigation as RT adjuncts to alleviate hypoxia and potentiate ROS-mediated killing.

Altogether, clinical data demonstrate that nanocarriers can cross the human BBB/BBTB, accumulate in GBM, and modulate molecular pathways or local energy deposition. However, survival gains remain modest, underscoring the need for better patient selection, mechanistically designed combinations (particularly with immunotherapy) and more precise, image-guided integration of physical-field activation.

## Current gaps, limitations, and challenges

Despite rapid progress, nanomedicine and physical-field–assisted strategies have not yet transformed GBM outcomes. The main barriers remain tumor heterogeneity, the BBB/BBTB, an immunosuppressive microenvironment, and the limited predictive value of current experimental models.

### Biological hurdles

GBM shows profound inter- and intratumoral heterogeneity at genetic, epigenetic, and phenotypic levels. Distinct subclones differ in proliferation, invasiveness, metabolic states, and expression of targets such as EGFR, PDGFR, and integrins. Glioma stem-like cells (GSCs) reside in hypoxic and perivascular niches that are poorly reached by drugs and relatively protected from physical insults.

For nanomedicine, this means receptor-targeted carriers may only address selected subpopulations, leaving antigen-low or antigen-negative clones to drive recurrence. Heterogeneous BBTB permeability and variable expression of efflux transporters (P-gp, BCRP) further generate uneven intratumoral exposure, so even well-designed BBB-targeting or biomimetic systems rarely achieve homogeneous drug distribution. Physical-field therapies face analogous constraints: regions with poor NP accumulation or limited light/field penetration are less affected, allowing regrowth from spared niches.

#### Immune evasion and adaptive resistance

GBM establishes a strongly immunosuppressive TME enriched in tumor-associated macrophages/microglia, regulatory T cells, and myeloid-derived suppressor cells, and characterized by high levels of TGF-β, IL-10, and PGE₂; PD-L1 upregulation further limits T ell activity. These features blunt the capacity of nanodrugs or field-induced immunogenic cell death (after PDT, PTT, or hyperthermia) to generate durable systemic antitumor immunity. Moreover, GBM cells exhibit therapy-induced plasticity, reprogramming metabolism and transiently increasing DNA repair and antioxidant defences (e.g., MGMT, glutathione systems), which reduces sensitivity to ROS-driven killing and undermines repeated treatment cycles [163].

Thus, even stimuli-responsive and biomimetic nanoplatforms risk only transient responses if they do not simultaneously reprogramme the immune microenvironment and counter adaptive resistance pathways.

### Technical and material challenges

Designing GBM nanomedicines requires balancing BBB penetration, tumor retention, drug loading, stability, biodegradability, and manufacturability, often with competing demands.

#### Instability, degradation, and clearance

Polymeric and lipid nanocarriers may degrade or leak cargo prematurely in blood, particularly after protein corona formation that masks targeting ligands and alters biodistribution. Inorganic or hybrid platforms (iron oxide, gold, hafnium) provide robust field responsiveness but can persist in the liver, spleen or brain, raising long-term toxicity concerns.

Ultrasmall NPs (< 5–6 nm) favor renal clearance but have limited drug-loading capacity and weaker photothermal/magnetic responses. Larger particles (50–150 nm) carry more payload and show stronger EPR-like accumulation but are more susceptible to RES uptake and long-term retention. Optimizing size, shape, and surface chemistry for both potent anti-GBM effects and predictable clearance therefore remains a central biomaterials design problem, particularly for responsive and biomimetic systems intended for chronic use.

#### Off-target effects and energy deposition

Even with active targeting, a large fraction of injected NPs is sequestered by the liver, spleen, and lungs. Overexpression of transport receptors (TfR, LDLR) in normal tissues can compete with tumor uptake. For field-based therapies, off-target NP accumulation is particularly problematic: local heating or ROS generation must be strictly confined to tumor regions to avoid collateral brain injury.

Precisely controlling energy deposition in the deep brain is technically demanding. Magnetic hyperthermia requires careful optimization of alternating magnetic field parameters; PTT/PDT depend on non-uniform light propagation; and FUS-induced cavitation is sensitive to skull anatomy and tissue interfaces. Real-time thermal and ROS monitoring is still limited clinically, complicating translation of preclinical dose response relationships.

### Limited predictability from preclinical models

Standard GBM models (2D cell lines, subcutaneous xenografts) fail to reproduce human BBB/BBTB, immune microenvironment, and spatial heterogeneity, often overestimating nanoparticle penetration and efficacy. Orthotopic xenografts and GEMMs better mimic growth patterns and BBTB features, but species differences in BBB transporters, immune responses, and clearance remain substantial. Emerging systems including patient-derived organoids, microfluidic BBB-on-chip devices, and ex-vivo brain-slice cultures offer more physiological contexts to study nanoparticle transport and field responses, but are not yet standardized, and their quantitative predictive power for human PK/toxicity is still being established. For next-generation nanomedicines, especially stimuli-responsive and biomimetic constructs, systematic validation across such complementary platforms will be essential before clinical translation.

#### Failures in clinical translation

Many GBM nanomedicines that show robust preclinical efficacy have failed to significantly extend survival in trials. Bridging this gap will require standardized preclinical pipelines that integrate BBB-relevant models, longitudinal multimodal imaging, immune readouts, and early consideration of manufacturability and regulatory expectations, aligned with the specific mechanisms of each nanoplatform (drug delivery vs. field activation vs. immune reprogramming) [206].

## Future perspectives and opportunities

The future development of GBM nanomedicine will depend less on introducing new carrier types and more on improving biological precision, translational predictability, and clinical manufacturability [207]. The most promising directions are (i) better alignment of nanocarriers with GBM molecular heterogeneity, (ii) integration of nanomedicine with image-guided and field-assisted delivery, and (iii) development of preclinical models that more faithfully reproduce human BBB/BBTB structure and tumor complexity [208].

In parallel, focused ultrasound, magnetic hyperthermia, and other externally controlled approaches may improve spatiotemporal selectivity when combined with BBB-aware and tumor-responsive nanoplatforms [209, 210].

### Artificial intelligence-guided nanoparticle design

AI and ML may help accelerate GBM nanomedicine design by linking formulation parameters with BBB penetration, tumor accumulation, release behavior, and toxicity [211]. In practical terms, these tools may reduce empirical trial-and-error during carrier optimization and improve formulation reproducibility during scale-up [212]. Their near-term value is likely to be greatest in formulation screening, parameter optimization, and patient stratification, rather than in fully automated nanomedicine design [4].

### Personalized nanomedicine approaches

Because GBM is highly heterogeneous, future nanotherapies will likely need to align targeting ligands and payloads with patient-specific molecular features such as EGFRvIII, IL–13Rα2, CD44, and treatment-resistance pathways [213–215]. The most realistic personalized direction is not a fully bespoke nanoparticle for each patient, but modular platforms that can be adapted to different biomarker-defined subgroups. Such an approach may improve selectivity while remaining compatible with clinical manufacturing [216, 217].

### Advanced navigation and real-time monitoring systems

Theranostic systems that combine drug delivery with MRI-, PET-, or fluorescence-based tracking may improve the clinical utility of GBM nanomedicine by showing where nanoparticles accumulate and when treatment should be activated [218–220]. In GBM, this is especially relevant for magnetic hyperthermia, focused ultrasound, and laser-based approaches, where treatment efficacy depends strongly on local delivery and controlled energy deposition. Future progress will depend on making these platforms not only multifunctional, but also reproducible, safe, and clinically practical [221].

## Conclusion

Glioblastoma (GBM) is an extremely challenging malignancy to treat because it is characterized by marked cellular heterogeneity and by the presence of the blood–brain barrier, which limits drug delivery. Although surgery, radiotherapy, and chemotherapy remain the standard treatment modalities, their impact on long–term survival remains limited. In this context, nanotechnology has emerged as a promising strategy to improve treatment precision, drug stability, and selective delivery to tumor tissue. Lipid-based nanoparticles, polymeric carriers, inorganic nanomaterials, and biomimetic nanoplatforms have demonstrated promising preclinical potential, particularly in terms of biocompatibility, transport efficiency, controlled delivery, and therapeutic specificity. Despite these advances, the clinical translation of GBM nanomedicines remains limited. Major barriers include inconsistent intratumoral delivery, biological heterogeneity, toxicity concerns, manufacturing complexity, and the limited predictive value of current preclinical models. Therefore, future progress will depend not only on the development of more sophisticated nanoplatforms, but also on improved model systems, better patient stratification, scalable formulation strategies, and mechanism-driven clinical evaluation. With continued interdisciplinary effort, nanomedicine may become an important component of more precise and effective GBM therapy.

## Data Availability

This review article does not generate new datasets. All data discussed are available in the cited literature and publicly accessible databases.

## Glossary

BBB: Blood–brain barrier, a specialized vascular interface regulating exchange between blood and CNS.
BBTB: Blood–brain tumor barrier, a heterogeneous and partially disrupted form of the BBB in and around GBM lesions.
GBM: Glioblastoma, a WHO grade IV diffuse astrocytic tumor and the most aggressive primary malignant brain tumor in adults.
NDDS: Nanomaterials–based drug delivery systems designed to improve pharmacokinetics and targeting of therapeutics.
PTT: Photothermal therapy, using light–absorbing agents to convert light into heat for tumor ablation.
PDT: Photodynamic therapy, using a photosensitizer plus light to generate cytotoxic reactive oxygen species.
